# Supplementary material for: Winter cancellations of elective surgical procedures in the UK: a questionnaire survey of patients on the economic and psychological impact
Source: BMJ Open. 2019 Sep 13;9(9):e028753. doi: 10.1136/bmjopen-2018-028753 (PMC6747666; doi:10.1136/bmjopen-2018-028753)
Supplement: Supplementary data [file bmjopen-2018-028753supp001.pdf]

emsan.co.uk/wespi wespistudy@gmail.com

Winter Elective Surgery Cancellation and Psychological Impact survey

WES-Pi

Please tell us about you:

Q1) What is your employment status?
*Please select **one** option only.*

- Employed - full time ☐
- Employed - part time ☐
- Self-employed - full time ☐
- Self-employed - part time ☐
- In education - full time ☐
- In education - part time ☐
- Unemployed ☐
- Retired ☐

Q2) What is your age group?
*Please select **one** option only.*

- 16-24 years ☐
- 25-34 years ☐
- 35-44 years ☐
- 45-54 years ☐
- 55-64 years ☐
- 65-74 years ☐
- 75-84 years ☐
- Over 85 years ☐
- Prefer not to say ☐

Q3) On what date was your operation meant to be?
Please provide an estimate if you are unsure of the exact date.

Date: d d - m m - y y y y

Q4) On what date were you told your operation was cancelled or postponed?
Please provide an estimate if you are unsure of the exact date.

Date: d d - m m - y y y y

Q5) If your operation was cancelled or postponed previously, how many times did this occur in total?
Please provide an estimate if you are unsure of the exact number of times.

[ ] times

CODE: [ ]

Herrod PJJ, *et al. BMJ Open* 2019; 9:e028753. doi: 10.1136/bmjopen-2018-028753

Q6) What operation were you meant to be having?
Please describe in your own words, e.g. hernia operation, hip replacement, etc.

Please tell us about the impact of your operation being cancelled or postponed on <u>your work and finances</u>:

Q7) Due to your operation being cancelled or postponed, were <u>you</u> able to **go to work** instead?
Please provide an estimate of the days you think you needed to take off work.

- Yes ☐
- No* ☐
- Not applicable ☐

*If not, how many **days off** did <u>you</u> take?

Q8) Do <u>you</u> think <u>you</u> will need **extra days off work** because your operation was cancelled or postponed?
Please provide an estimate of the day/s you think you will need off work, due to your untreated condition preventing you from working, or the additional time off you will have to take when you receive another date for your operation.

- Yes* ☐
- No ☐
- Not applicable ☐

*If yes, how many **day/s off**?

Q9) Did your <u>friends or family members</u> have to take **extra days off work** because your operation was cancelled or postponed?
Please provide an estimate of the day/s your friends or family members have taken so far, to either look after you when unwell (because you did not have your operation), or planned to take off to look after you following your operation

- Yes* ☐
- No ☐
- Not applicable ☐

*If yes, how many **day/s off**?

Q10) If you are a parent or carer, have <u>you</u> needed **extra childcare** or **carer support**, because your operation was cancelled or postponed?

Please provide an estimate of the total time you have needed so far. e.g. having to book extra support when you are unwell (because you did not have your operation), or needing to rebook extra support for when you receive another operation date.

Yes* ☐
No ☐
Not applicable ☐

*If yes, how many **hours*** or **days***?

****please circle as appropriate***

Q11) Have <u>you</u> had **extra costs in travel and parking** because your operation was cancelled or postponed?

Please provide an estimate if you are unsure of the exact costs so far. e.g. additional journeys to the hospital/doctors because you have been unwell (because you did not have your operation), or because you have needed extra tests before receiving another operation date.

Yes* ☐
No ☐
Not applicable ☐

If yes, how much **extra cost**?

Please tell us about the impact of your operation being cancelled or postponed on your <u>mental health and well-being</u>:

Q12) Are there any key **social activities/hobbies** <u>you</u> have had to miss because your operation was cancelled or postponed?

These may include family days out, attendance at weddings, visiting friends, or your ability to physically participate in your hobbies or interests outside of work.

Yes* ☐
No ☐
Not applicable ☐

*If yes, is there anything specific you would like to mention?

Herrod PJJ, *et al. BMJ Open* 2019; 9:e028753. doi: 10.1136/bmjopen-2018-028753

For each question please select **one** option that best describes **your feelings, at the time** your operation was cancelled or postponed:	**Not at all**	**Slightly**	**Moderately**	**Very**	**Extremely**	**Not applicable**
Q13) How **sad** were you?	☐	☐	☐	☐	☐	☐
Q14) How **disappointed** were you?	☐	☐	☐	☐	☐	☐
Q15) How **angry or annoyed** were you?	☐	☐	☐	☐	☐	☐
Q16) How **frustrated** were you?	☐	☐	☐	☐	☐	☐
Q17) How **stressed** were you?	☐	☐	☐	☐	☐	☐

For each question please select **one** option that best describes **your concerns, at the time** your operation was cancelled or postponed:	**Not at all**	**Slightly**	**Moderately**	**Very**	**Extremely**	**Not applicable**
Q18) How concerned were you about your **symptoms continuing**?	☐	☐	☐	☐	☐	☐
Q19) How concerned were you about your **health deteriorating**?	☐	☐	☐	☐	☐	☐

Q20) Is there anything else you would like to mention about how your operation being cancelled or postponed has affected you?
e.g. how it made you feel, how it impacted on your life, how it affected you physically, whether you were given enough information, etc.

Thank you for taking the time to complete this survey

Please return it in the pre-paid envelope provided within **two weeks** of receipt.

CODE: [ ]
