# Supplementary material for: Winter cancellations of elective surgical procedures in the UK: a questionnaire survey of patients on the economic and psychological impact
Source: BMJ Open. 2019 Sep 13;9(9):e028753. doi: 10.1136/bmjopen-2018-028753 (PMC6747666; doi:10.1136/bmjopen-2018-028753)
Supplement: Supplementary data [file bmjopen-2018-028753supp002.pdf]

NHS
National Institute for Health Research

WES-Pi

1st March 2018

Dear Patient,

As you may be aware, due to the increased pressures faced by the NHS during the winter period, some patients like yourself have unfortunately had their operations either cancelled or delayed.

To understand the true impact this can have on our patients' lives, we are asking all patients who have experienced a cancellation or delay to complete the attached short survey (Winter Elective Surgery Cancellation and Psychological Impact survey).

You do not have to participate in this survey, but your experiences may help us to improve our services for future patients. There are no right or wrong answers to any of the questions asked, it is your opinions that we are really interested in. In particular, we would like to hear how your experience of a cancellation has affected you emotionally, physically, and financially.

You do not have to give any personal details, and the results of the survey will be completely anonymous when published. To participate, please fill in the attached survey and return it to us in the pre-paid envelope provided. If you have any further questions about this survey you can contact us at: wespistudy@gmail.com

Thank you for your time.

Yours sincerely,

The East Midlands Surgical Academic Network (EMSAN)

EMSAN is a group of trainee doctors who work for the NHS. We believe in making research opportunities available to everyone, to aid learning and improve medical services.

Visit us at: emsan.co.uk/wespi
Contact us at: wespistudy@gmail.com

Patient Public Involvement in study survey design

Question No.	Question	PPI Lay Assessors – random comments	Study lead response
Q5	In your own words, what operation were you meant to be having?	Wording change: "Had your operation been previously cancelled or postponed? If so, how many times?"	Amended
Q8	How many **extra** days off work do you now think **you** will miss because of the health condition that you were due to have the operation for?	This is a rhetorical question I doubt even Mr Adiamah could answer correctly.	This has now been amended to reflect the time taken for the surgery and immediately afterwards, and potential time taken off due to ongoing illness related to the cancellation
Q10	How much extra cost has **childcare** cost you because of your operation being cancelled or postponed?	Question on childcare costs. May not be relevant to all participants, and potentially quite intrusive. Also, costs can be very variable.<br><br>Suggestion of using 'hours of childcare' instead.	Question amended to ask about 'hours of childcare<br><br>AND carer support' if relevant.<br><br>Descriptor added.
Q13-Q18	Please answer the following questions in relation to your operation being cancelled or postponed.<br><br>*For each question please select ONE option that*	Surely 'today' might not be a good measure of feelings about the change in plan	Amended to ask about feelings at the time of the postponement instead of 'today'.

	best describes your feelings ***today.*** <br>How **disappointed (Q13); angry (Q14); frustrated (Q15); stressed (Q16)** are you about your operation? <br>How concerned are you about <br>your **symptoms continuing**? (Q17); **health deteriorating**? (Q18)		
Overall	Additional box for other comments (this was suggested by two of the PPI Lay Assessors)		Great suggestion, thanks – we have added this.
