# Supplementary material for: Winter cancellations of elective surgical procedures in the UK: a questionnaire survey of patients on the economic and psychological impact
Source: BMJ Open. 2019 Sep 13;9(9):e028753. doi: 10.1136/bmjopen-2018-028753 (PMC6747666; doi:10.1136/bmjopen-2018-028753)
Supplement: Author's manuscript [file bmjopen-2018-028753.draft_revisions.pdf]

BMJ Open

BMJ Open is committed to open peer review. As part of this commitment we make the peer review history of every article we publish publicly available.

When an article is published we post the peer reviewers' comments and the authors' responses online. We also post the versions of the paper that were used during peer review. These are the versions that the peer review comments apply to.

The versions of the paper that follow are the versions that were submitted during the peer review process. They are not the versions of record or the final published versions. They should not be cited or distributed as the published version of this manuscript.

BMJ Open is an open access journal and the full, final, typeset and author-corrected version of record of the manuscript is available on our site with no access controls, subscription charges or pay-per-view fees (http://bmjopen.bmj.com).

If you have any questions on BMJ Open's open peer review process please email info.bmjopen@bmj.com

Winter cancellations of elective surgical procedures in the United Kingdom: A questionnaire survey of patients on the economic and psychological impact

Journal:	*BMJ Open*
Manuscript ID	bmjopen-2018-028753
Article Type:	Research
Date Submitted by the Author:	21-Dec-2018

Complete List of Authors:	Herrod, Philip; Royal Derby Hospital, Department of Surgery<br>Adiamah, Alfred; Need for Nutrition Education Programme group,<br>Boyd-Carson, Hannah; Royal Derby Hospital, Derby, UK DE22 3NE, Department of Surgery<br>Daliya, Prita; Nottingham University Hospitals, Queens Medical Centre, Gastrointestinal Surgery<br>El-Sharkawy, Ahmed; Royal Derby Hospital,<br>Sarmah, Panchali; University Hospitals of Leicester NHS Trust<br>Hossain, Tanvir; Nottingham University Hospitals, Queens Medical Centre, Gastrointestinal Surgery<br>Couch, Jennifer; Nottingham University Hospitals NHS Trust, Department of General Surgery<br>Sian, Tanvir; Royal Derby Hospital, Department of Surgery<br>Wragg, Andrew; Nottingham University Hospitals NHS Trust, Patient And Public Initiative Facilitator<br>Andrew, David; Lincoln County Hospital<br>Parsons, Simon; Nottingham University Hospitals NHS Trust, Department of General Surgery<br>Lobo, Dileep; Nottingham University Hospitals, Queens Medical Centre, Gastrointestinal Surgery

Keywords:	SURGERY, Adult surgery < SURGERY, QUALITATIVE RESEARCH, Organisation of health services < HEALTH SERVICES ADMINISTRATION & MANAGEMENT

SCHOLARONE™
Manuscripts

Winter cancellations of elective surgical procedures in the United Kingdom: A questionnaire survey of patients on the economic and psychological impact

PJJ Herrod*[1,2], A Adiamah*[1,2], H Boyd-Carson[1,2], P Daliya[1,2], AM El-Sharkawy[1,2], PB Sarmah[1,3], T Hossain[1,2], J Couch[1,2], TS Sian[1,2], A Wragg[4], DR Andrew[5], SL Parsons[1,2], DN Lobo[1,2,6], WES-Pi Study Group on behalf of the East Midlands Surgical Academic Network†

*Joint first authors

[1]East Midlands Surgical Academic Network, Queen's Medical Centre, NG7 2UH, UK
[2]Gastrointestinal Surgery, Nottingham Digestive Diseases Centre and National Institute for Health Research (NIHR) Nottingham Biomedical Research Centre, Nottingham University Hospitals and University of Nottingham, Queen's Medical Centre, Nottingham NG7 2UH, UK
[3]Leicester Cancer Research Centre, University of Leicester, LE2 7LX, UK
[4]Patient & Public Involvement Facilitator, Gastrointestinal and Liver Disorders Theme, National Institute for Health Research (NIHR) Nottingham Biomedical Research Centre, Nottingham University Hospitals NHS Trust and the University of Nottingham, Queen's Medical Centre, Nottingham NG7 2UH, UK
[5]Lincoln County Hospital, Greetwell Road, Lincoln LN2 5QY, UK
[6]MRC/ARUK Centre for Musculoskeletal Ageing Research, School of Life Sciences, University of Nottingham, Queen's Medical Centre, Nottingham NG7 2UK, UK

† WES-Pi Study Group:

Jennifer Couch, Prita Daliya, Tanvir Hossain, Bethan Johnson, Amanda Koh, Anisa Kushairi, Dileep N Lobo, Simon L Parsons, Andrew Wragg (Nottingham University Hospitals NHS Trust); Alfred Adiamah, David R Andrew, Chris Lewis-Lloyd, Farah Roslan, Amari Thompson, Helen Wan (United Lincolnshire Hospitals NHS Trust); Ahmed M El-Sharkawy, Sita Kotecha, Kevin Sargen (Chesterfield Royal Hospitals NHS Foundation Trust); Hannah Boyd-Carson, Phillip J J Herrod, Jonathan N Lund, Alastair Morton, Tanvir S Sian (Royal Derby NHS Foundation Trust); Edmund Charles, Panchali B Sarmah, Baljit Singh (University Hospitals of Leicester NHS Trust).

Address for correspondence:
Professor Dileep N Lobo
Gastrointestinal Surgery
Nottingham Digestive Diseases Centre
Nottingham University Hospitals NHS Trust and University of Nottingham
E Floor, West Block
Queen's Medical Centre
Nottingham NG7 2UH, UK
Tel: +44-115-8231149
Fax: +44-115-8231160
E-mail: Dileep.Lobo@nottingham.ac.uk

For peer review only

Category: Original research

Word count (excluding title page, abstract, figure legends, tables and references): 2961

Table and figures: 4 tables and 3 figures + 1 supplementary digital document

Running head: Winter Elective Surgery Cancellation and Psychological Impact (WES-Pi) Survey

Key words: winter cancellations; elective operations; psychosocial impact; economic impact; National Health Service

Funding: This work was supported by the Medical Research Council [grant number MR/K00414X/1]; and Arthritis Research UK [grant number 19891]. AA was funded by a National Institute for Health Research (NIHR) Academic Clinical Fellowship.

The funders had no role in the design or conduct of the work, or in the decision to publish. This paper presents independent research funded by the MRC, ARUK and the NIHR. The views expressed are those of the authors and not necessarily those of the MRC, ARUK, NHS, the NIHR or the Department of Health.

Conflict of interest: None of the authors has a direct conflict of interest to declare. SLP is a company director for EIDO Healthcare Limited (unrelated to the submission). DNL has received an unrestricted research grants for unrelated work from BBraun in the last 3 years. He has also received speakers' honoraria from BBraun, Fresenius Kabi, Shire and Baxter Healthcare for unrelated work in the last 3 years.

This paper will be presented to the Annual Meeting of the Society for Academic and Research Surgery in London, UK on 8 January 2019. It will be published in abstract form in the *British Journal of Surgery*.

"The Corresponding Author has the right to grant on behalf of all authors and does grant on behalf of all authors, a worldwide licence to the Publishers and its licensees in perpetuity, in all forms, formats and media (whether known now or created in the future), to i) publish, reproduce, distribute, display and store the Contribution, ii) translate the Contribution into other languages, create adaptations, reprints, include within collections and create summaries, extracts and/or, abstracts of the Contribution, iii) create any other derivative work(s) based on the Contribution, iv) to exploit all subsidiary rights in the Contribution, v) the inclusion of electronic links from the Contribution to third party material where-ever it may be located; and, vi) licence any third party to do any or all of the above."

For peer review only

ABSTRACT

Objectives: To quantify the economic and psychological impact of cancellation of operations due to winter pressures on patients, their families and the economy.

Design: This questionnaire study was designed with the help of patient groups. Data were collected on the economic and financial burden of cancellations. Emotions were also quantified on a 5-point Likert scale.

Setting: Five NHS Hospital Trusts in the East Midlands region of England.

Participants: We identified 796 participants who had their elective operations cancelled between 1-11-2017 and 31-3-2018 and received responses from 339 (43%).

Interventions: Participants were posted a modified version of a validated quality of life questionnaire with a pre-paid return envelope.

Main Outcome Measures: The primary outcome measure was the financial and psychological impact of cancellation of elective surgery on patients and their families.

Results: Of the 349 respondents, 163 (48%) were aged <65 years, with 111 (68%) being in employment. Sixty-six (19%) had their operations cancelled on the day. Only 69 (62%) of working adults were able to return to work during the time scheduled for their operation, with a mean (standard deviation, SD) loss of 5 working days (SD 10). Additional working days were lost subsequently by 60 (54%) participants [mean 7 days (SD 10)]. Family members of 111 (33%) patients required additional time off work [mean 5 days (SD 7)]. At least moderate concern about continued symptoms was reported by 234 (70%) participants whilst awaiting

For peer review only

rescheduling, and 193 (59%) reported at least moderate concern about their condition deteriorating.

Conclusions: The mass cancellation of elective surgery during the winter had a significant adverse impact on patients and the economy, including days of work lost and health-related anxiety.

For peer review only

Strengths

- This study presents aggregated evidence to support previous anecdotes of the importance of cancellation of elective operations to patients' well-being. It was devised with active consultation with patient groups. The questions asked were those that the patient groups thought relevant to the problem.
- It highlights the importance of early notification of cancellations in times such as winter pressures, where cancellations are unavoidable to sustain the delivery of emergency care.

Limitations

- This study was retrospective by design and was potentially limited by recall bias
- We achieved a less than 50% responder rate, in spite of two rounds of questionnaires being sent, and as such is subject to non-responder bias.
- The study covered a single but large region of the United Kingdom, it is difficult to ascertain if the population dynamics and level of social deprivation make this population similar to those in all parts of the UK, and as such affects the generalisability of our results.

For peer review only

INTRODUCTION

The winter of 2017-2018 saw an unsustainable increase in patient demand on acute hospital care in England, due both to an anticipated seasonal rise in admissions and an unexpectedly severe epidemic of winter flu. In response to what was dubbed the "Winter National Health Service (NHS) Crisis" by the media, NHS England took the unprecedented step of instructing all NHS hospital trusts to cancel planned routine non-cancer surgery throughout December 2017 and January 2018, in order to free resources for emergency admissions.[1]

Although official figures have not yet been released, media estimates put the number of patients affected in England at around 50,000.[2] This huge volume of cancellations will have ongoing ramifications for the 2018 calendar year. The rescheduling of a large backlog of cancelled operations by hospital trusts, will undoubtedly put added pressure on hospital waiting lists.

Although, the effect of a cancellation on the ongoing care provided by hospitals is subject to many reporting structures, the psychological and economic impact of a cancellation on patients themselves has received remarkably little attention. The requirement of a period of recuperation, means most patients in their preparation for elective surgery make arrangements in their social lives to facilitate this. Adults in employment may book time off work and postpone important social events such as holidays. Carers may similarly organise additional support or childcare arrangements. Family members may also do the same in order to help care for recovering patients. If a planned operation is postponed at short notice, it is possible to envisage these arrangements resulting in personal and economic loss.

For peer review only

Any rescheduled operative date may also result in patients and carers incurring the same costs again with no recourse to reimbursement. This may lead to a considerable economic burden on the patient and their family[3] and cause additional loss of working days for the economy. Postponement or cancellation of elective surgery may also have a profound psychological impact on patients and their families, who may have mentally prepared themselves for the event. There may also be significant health-related anxiety associated with a longer wait for surgery, as patients may fear their condition deteriorating further. We aimed, in this **W**inter **E**lective **S**urgery Cancellation and **P**sychological **i**mpact (WES-Pi) survey, to quantify the financial and psychological impact on patients following the postponement or cancellation of elective surgery in England, due to winter pressures.

For peer review only

METHODS

This multicentre study was conducted across five NHS acute hospital trusts in the East Midlands region of England during the winter of 2017-18. Adult patients aged 16 years or over, scheduled for elective surgery between 1 November 2017 and 31 March 2018 were identified from local hospital waiting list records. Participants were chosen from the surgical subspecialties of general surgery, colorectal, hepatopancreaticobiliary, upper gastrointestinal, endocrine, vascular, urology, orthopaedics, maxillofacial, and ear nose and throat. All patients who had their elective surgery postponed or cancelled for non-patient related reasons were included (**Figure 1**). Patients who had surgery postponed or cancelled for patient-related reasons (e.g. unrelated illness, medically unfit, or surgery declined), or those that died before dispatch of the questionnaires were not invited to participate.

Outcomes

Our primary outcome measure was to quantify the financial and psychological impact of cancelation of elective surgery on participants and their families. The secondary outcome measure was to evaluate the impact of the length of notice of cancellation on the primary outcomes.

Survey design

In the absence of an existing validated questionnaire to explore financial or emotional consequences to a patient, specifically related to the cancellation of elective surgery, we

For peer review only - http://bmjopen.bmj.com/site/about/guidelines.xhtml

modified a previously used survey from a paediatric study.[4] Survey design involved multiple iterations and amendments through patient focus groups and significant involvement and input from the Patient Advisory Group at Nottingham University Hospitals NHS trust, and the Patient Advisory and Liaison Service (PALS) team at Derby Teaching Hospitals NHS Trust. In addition to gathering data on the economic and financial burden of postponements and cancellations on participants and their relatives, the questionnaire also quantified emotional feelings of participants on a 5-point Likert scale[5] (**Table 1**).

Patient and public involvement

Patient & Public Involvement (PPI) was an integral element throughout this study given its direct relevance to patient care. Embedding PPI into to the study from the outset meant we could more accurately explore the patient experience of cancellations informed by what patients felt from prior experience were important in their care. There is a current drive for PPI in all medical research, for experiential reasons (Patients or carers have experience of a condition, treatment or in this instance experience of a cancelled appointment for elective operation); ethical and moral reasons (Patients and the public are directly affected by the research process and findings; they have the right to contribute to it; and of course are the tax payers); and pragmatic reasons (PPI is a requirement for funding bodies; it translates research into knowledge). In this instance, we felt strongly that for a study where the purpose was solely about patient experience, it had to be informed fully by patients.

We chose to recruit PPI Lay Assessors to review the invitation letter and subsequent survey to be sent to study participants. We wanted to prove that the intent to use a survey to explore the impact of cancelled elective operations on the psychosocial wellbeing of our

For peer review only

patients, as a means leading to service improvement, if suitably structured would achieve its outcomes. PPI provided assurance that the devised series of questions contained in the survey was suitable to cover the aspects of financial, psychological and physical impact of these cancellations including symptomatic (suggestive) deterioration without adversely impacting on patients or being too onerous to complete.

They directed the changes through several iterations and approved of the final survey and invitation letter, ensuring the overall suitability and acceptability of the survey.

How was the development of the research question and outcome measures informed by patients' priorities, experience, and preferences?

In consultation with PPI members, the research tool was shaped to more accurately assess the financial impact of cancellations, not only for patients but also for their family members involved in their care. In exploring patients' emotions, their responses allowed us to make the questions more specific to feelings at the time of cancellations. See attached appendix 1

How did you involve patients in the design of this study?

From the PPI perspectives, the measures were informed via six (6) recruited PPI members from the Gastrointestinal & Liver Patient Advisory Group, charged with reviewing these study public-facing documents (invitation letter and survey to be issued to the study participants). Each lay PPI Member assessed and reported back their respective thoughts on the document(s) content, layout and its message. Examples of comments (on the survey) made, and responses made by Study leads are presented in Appendix 1

For peer review only - http://bmjopen.bmj.com/site/about/guidelines.xhtml

Were patients involved in the recruitment to and conduct of the study?

No. Recruitment was informed by the cancellation list at each participating trust.

How will the results be disseminated to study participants?

The write up of the manuscript included the lead person from the GI PPI group, allowing the patient viewpoints to be more accurately reflected in our results. It also ensures dissemination to the initial group who were involved in the study design. More importantly there is an opportunity for this study to aid advocacy in the conduct of patient cancellations in a manner that results in the least adverse outcomes for patients.

For randomised controlled trials, was the burden of the intervention assessed by patients themselves?

Not applicable.

Data collection

The study was registered and approved locally at each participating hospital trust, under the supervision of a named consultant surgeon. Identified participants were posted a copy of the study questionnaire (Table 1), a pre-paid return envelope, and an invitation letter (Supplementary Data), incorporating local trust branding. Invitations were first posted to participants in April 2018, with a further invitation to non-responders in June 2018. Each study site was managed by local teams responsible for local study registration and approvals,

For peer review only

identification of participants, dissemination of postal invitations, and data collection. Returned surveys were pseudoanonymised, with collated data subsequently anonymised and encrypted before central data analysis. No identifiable participant data were transferred between sites.

Data synthesis

Quantitative and qualitative data analyses of responses were undertaken. Quantitative data analysis was performed using STATA® v15.0 (StataCorp, College Station, TX, USA). Linear regression was performed to assess for correlation. Qualitative data was evaluated using NVivo v11 (QSR International Inc., Burlington, MA, USA) for thematic analysis. All descriptive data are represented as n (%), mean (standard deviation, SD) or median (inter quartile range, IQR) as appropriate.

For peer review only

RESULTS

Return rate and cancellation

Questionnaires were posted to 796 participants and returned by 339 (43%) after two distribution rounds. The response rate varied between trusts from 27% to 52%. Data on participant characteristics and surgical specialties involved are displayed in **Table 2**.

Of the participants returning questionnaires, 66 (19%) had their operations cancelled on the day of surgery, 44% (n=149) within 24 hours of the proposed surgery, and 37% (n=125) cancelled more than 48 hours from the proposed date of the operation. Median notification of cancellation was 2 days (IQR 1-6). A number of participants had been cancelled previously prior to the winter cancellation. The responders reported their operation as having been cancelled a median of 1 time (IQR 1-2) previously. In 15% (45/288) of the responders, operations had been cancelled three times or more (**Table 2**).

Economic burden

One hundred and sixty-three (48%) of responding participants were aged less than 65 years and of these 111 (68%) were in employment. Only 69 (62%) of the working adults were able to return to work during the time they had originally scheduled off for their operation, with a median loss of 1 day (IQR 0-5) of work. The total number of work days lost in the cohort was 329 (**Figure 2**).

Additional working days in subsequent months were lost by 60 (54%) of working adults due to the cancellation [median 3 days (IQR 0-12)], totalling 456 days. Family members of 111

For peer review only

(33%) participants required additional time off work median 3 days (IQR 1-5), totalling 581 days. Thus, the total number of missed days of work (from participants and family members) was 1366 days. There was no direct correlation between days of work lost and the notification given for cancellation (r^2=0.0004, p=0.87).

Child care costs

Of the responders with children, 23 were forced to organise extra childcare. Of these the median (IQR) additional childcare days required was 4 days (0-22.5).

Travel costs

One hundred and twenty-three participants (48%) incurred additional travel costs following cancellation of their operation. The median cost incurred for additional travel was £15 (IQR £6.5-£30).

Psychological burden

The majority of participants reported at least moderate levels of sadness, disappointment, anger, frustration and stress, with over 30% (n=102) of respondents reporting extreme levels of each of these emotions. A full breakdown of emotions reported is displayed in **Table 3**. At least moderate concern about continued symptoms was reported by 234 (70%) patients

For peer review only

whilst waiting for a rescheduled date whilst 193 (59%) reported at least moderate concern about their condition deteriorating.

There was no correlation between strength of any emotion and the amount of notice given to participants for the cancellation. This was true for all the emotive questions asked.

Thematic analysis from free text responses

The qualitative analysis of the participants' free text responses is presented in the thematic analysis as a radar diagram (**Figure 3**). Four particular themes were highlighted in this analysis. There was concern over symptoms worsening; stress and anxiety related to the cancellation; and those whose operations were cancelled on the day felt that this was unsatisfactory and caused them undue distress. However, the most interesting finding on the free text analysis was that 26% (70/268) of the responders understood the need for their operations to be cancelled to facilitate delivery of the emergency service. They used such descriptions as: "my operation was not life-threatening" and "others needed the care more" (**Table 4**).

For peer review only

DISCUSSION

The uncharacteristically cold weather, the high rates of emergency admissions, particularly in the elderly, and a lack of capacity in an already overworked and overstretched system gave rise to the perfect storm of cancellations during the winter of Nov 2017 to Feb 2018. Some even referred to it as another winter of discontent for the NHS.[6] The national strategy, instigated by NHS England was to cancel elective operations to release capacity in the system.[1] These elective cancellations were well publicised in the media, and meant that the majority of the population had some idea of the unusual stress the NHS was under.[2 6] However, despite public awareness, the impact on patients of these cancellations and, in particular last minute cancellations, was significant.

What this study found

This study is the first in England to evaluate the impact of the mass cancellation of elective surgery on patients, from both an economic and psychological view point. We have demonstrated that a large proportion of patients suffered a negative economic impact from both the additional work days lost and the additional non-refundable travel and childcare costs. We achieved this using a novel research tool, developed jointly with patient groups to establish usability, acceptability and internal validity.

Taken in the context of a nation-wide bed pressure crisis, which was widely publicised in the media, the fact that nearly 20% of patients had their operations cancelled on the day of surgery was unacceptable. Despite this we were unable to demonstrate any correlation between the period of notice given for cancellation of the planned operation, and the number of working days lost, nor any impact on the responses that patients gave to the

For peer review only

questions on their emotional state. Although the reasons behind this are unclear, our study may have been underpowered to demonstrate this and also due to the over representation of retired patients in our cohort.

Our relatively small sample from one region of the UK, comprising 5 hospitals, contained nearly 800 cancelled operations and in those responders led to the loss of 1366 work days to the economy from our modest 43% response rate. Given that this crisis stretched to the whole length of the UK and was estimated to have led to 50,000 cancellations nationwide, the projected loss of working days may exceed 85,000 days, even on the unlikely assumption that the non-responding patients lost no days from work. The questions on psychological impact, demonstrated that elective surgical cancellation has led to a large amount of negative emotion amongst patients, with the majority displaying at least a moderate amount of health-related anxiety.

Although this study was about winter cancellations of operations of patients from one region of England, the results are probably generalisable to the rest of the UK and also to other countries. Moreover, the results are not just applicable to winter cancellations, but could be applied to cancellations occurring at other times of the year as well.

What is available in the literature?

Whilst several studies have evaluated the causes of bed pressures and failures of hospital trusts to meet the emergency department 4-hour performance target,[7 8] very little information is available on the psychosocial impact of cancellations of elective operations on patients. Others have reported on measures to reduce same day cancellations of elective operations, but have focused on other aspects, such as ensuring fitness for surgery,

For peer review only

preventing the scheduling of the wrong operation, or preventing incomplete preoperative assessments rather than addressing cancellations due to a lack of beds.[9] Others have counted the cost of such last minute cancellations to the hospital itself, from specialised equipment that might need to be brought in, and noted that cancellations within 24 hours impacted significantly on costs to the trust.[10] In this study we found that 19% of the patients had their operations cancelled within 24 hours. Whilst this resulted in a financial impact on the patients, such late cancellations are also likely to increase costs for the hospital trust itself. From the point of view of optimal deployment of personnel and resources, better planning is needed for future crises. The Royal College of Surgeons of England has published guidance to help manage winter pressures and has acknowledged that it was better for patients to be told in advance of their cancellations in agreement with the findings of this study.[11] Additionally, they acknowledged the consequences of such cancellations inevitably would include prolonged suffering of the patients affected.[11] Better forecast and long-term planning of population health needs by policy makers are the only ways to avert these winter cancellations from becoming a recurring theme in the delivery of healthcare in the UK.

Limitations

Our study may be limited by its response rate, potential recall bias and non-responder bias. We took steps to maximise our response rate by sending pre-paid return envelopes with each questionnaire, and by dispatching a second round of questionnaires to non-responders. However, despite these efforts we were unable to improve our response rate above 50%. Even on the unlikely assumption that the non-responders did not experience any negative

For peer review only

emotion or loss of work, the economic and psychological burden on this cohort was considerable.

For peer review only

CONCLUSIONS

The WES-Pi survey has provided evidence to support the often-cited anecdotes concerning the impact of cancelling elective operations on patients' finances and psychological wellbeing. The national awareness of cancellations meant that the majority of responding patients understood the need for these cancellations. However, with 20% of respondents having their operations cancelled on the day of the procedure better planning is needed in the future to reduce cancellations and to provide more notice and better support to patients who have their operations cancelled. This may require a national change in policy by the Department of Health, in collaboration with specialist clinical commissioning groups and local trusts.

For peer review only

REFERENCES

1. Campbell D, Duncan P. NHS cancels surgery for tens of thousands to avoid winter crisis. *The Guardian* 21 December 2017. Available at https://www.theguardian.com/society/2017/dec/21/nhs-cancels-surgery-tens-of-thousands-avoid-winter-crisis Accessed 10 November 2018.

2. Donnelly L, Bodkin H. NHS hospitals ordered to cancel all routine operations in January as flu spike and bed shortages lead to A&E crisis. *The Telegraph* 2 January 2018. Available at https://www.telegraph.co.uk/news/2018/01/02/nhs-hospitals-ordered-cancel-routine-operations-january/ Accessed 10 November 2018.

3. Lankoandé M, Bonkoungou P, Ki BK, et al. Economic and psychological burden of scheduled surgery cancellation in a sub-Saharan country (Burkina Faso). Southern African Journal of Anaesthesia and Analgesia 2017;**23**:145-51.

4. Tait AR, Voepel-Lewis T, Munro HM, Gutstein HB, Reynolds PI. Cancellation of pediatric outpatient surgery: economic and emotional implications for patients and their families. J Clin Anesth 1997;**9**:213-9.

5. Sullivan GM, Artino AR, Jr. Analyzing and interpreting data from likert-type scales. J Grad Med Educ 2013;**5**:541-42.

6. Campbell G. WINTER OF DISCONTENT? Plan to avoid hospital crisis at Christmas as pressure on A&E increases. *Oxford Mail* 17 November, 2017. Available at https://www.oxfordmail.co.uk/news/15666401.winter-of-discontent-plan-to-avoid-

For peer review only

hospital-crisis-at-christmas-as-pressure-on-ae-increases/ Accessed 10 November 2018

7. Keogh B, Culliford D, Guerrero-Luduena R, Monks T. Exploring emergency department 4-hour target performance and cancelled elective operations: a regression analysis of routinely collected and openly reported NHS trust data. BMJ Open 2018;**8**:e020296.

8. Morris AJ, McAvoy J, Dweik D, Ferrigno M, Macario A, Haisjackl M. Cancellation of elective cases in a recently opened, tertiary/quaternary-level hospital in the Middle East. Anesth Analg 2017;**125**:268-71.

9. McKendrick DR, Cumming GP, Lee AJ. A 5-year observational study of cancellations in the operating room: Does the introduction of preoperative preparation have an impact? Saudi J Anaesth 2014;**8**(Suppl 1):S8-S14.

10. Sanjay P, Dodds A, Sanjay P, Miller E, Arumugam PJ, Woodward A. Cancelled elective operations: an observational study from a district general hospital. J Health Organ Manag 2007;**21**:54-8.

11. Royal College of Surgeons of England. Guidance to help manage winter pressures. 28 November 2018. Available at https://www.rcseng.ac.uk/news-and-events/news/archive/guidance-to-help-manage-winter-pressures/ Accessed 12 December 2018.

For peer review only

Statements

Competing interest statement

All authors have completed the Unified Competing Interest form at http://www.icmje.org/coi_disclosure.pdf (available on request from the corresponding author) and declare: no support from any organisation for the submitted work; no financial relationships with any organisations that might have an interest in the submitted work in the previous three years, no other relationships or activities that could appear to have influenced the submitted work." Please see http://resources.bmj.com/bmj/authors/editorial-policies/competing-interests

Exclusive licence

The Corresponding Author has the right to grant on behalf of all authors and does grant on behalf of all authors, an exclusive licence (or non exclusive for government employees) on a worldwide basis to the BMJ Publishing Group Ltd and its Licensees to permit this article (if accepted) to be published in BMJ editions and any other BMJPGL products and sublicences to exploit all subsidiary rights, as set out in our licence)."

Contributorship statement

Study design: PJJH, AA, HBC, PD, AME-S, PBS, TH, JC, TSS, DRA, AW, SLP, DNL

Development of questionnaire: AA, PJJH, PD, HBC, AW

Data collection: PJJH, AA, HBC, PD, AME-S, PBS, TH, JC, TSS, DRA, AW, SLP, DNL

Data-analysis: PJJH, AA, HBC

Data-interpretation: PJJH, AA, HBC, PD, DNL

Writing of Manuscript: PJJH, AA, PD, HBC, DNL

Creation of figures: AA, PD, DNL

Critical review of Manuscript: DRA, AW, SLP, DNL

Final approval: PJJH, AA, HBC, PD, AME-S, PBS, TH, JC, TSS, DRA, AW, SLP, DNL

PPI member’s acknowledgements

We are very grateful to the following PPI members from the Gastrointestinal & Liver Patient Advisory Group, who worked on the questionnaire tool and whose contributions overall

For peer review only

shaped the study design. They are: Karen Andrews; David Harrison; David Matthews; John Shaw; Sally Taylor; and Stevie Vanhegan.

Guarantor Statement

The lead author (the manuscript's guarantor) affirms that the manuscript is an honest, accurate, and transparent account of the study being reported; that no important aspects of the study have been omitted; and that any discrepancies from the study as planned (and, if relevant, registered) have been explained.

Ethical approval

The study was registered and approved locally at each participating hospital trust, under the supervision of a named consultant surgeon.

Data sharing

Data will be available from the lead author.

Patient involvement

The survey design involved multiple iterations and amendments through patient focus groups and significant involvement and input from the Patient Advisory Group at Nottingham University Hospitals NHS trust, and the Patient Advisory and Liaison Service (PALS) team at Derby Teaching Hospitals NHS Trust. One of the authors (AW) is a Patient & Public Involvement Facilitator.

For peer review only

LEGENDS FOR FIGURES

Figure 1: Study flow diagram.

Figure 2: Number of work days lost.

Figure 3: Radar diagram of thematic analysis (figures represent percentage of responders).

For peer review only

Table 1: Survey proforma

emsan.co.uk/wespi   wespistudy@gmail.com

Winter Elective Surgery Cancellation and Psychological Impact survey

WES-Pi

Please tell us about you:

Q1) What is your employment status?
*Please select **one** option only.*

Employed	- full time	☐	In education	- full time	☐
	- part time	☐		- part time	☐
Self-employed	- full time	☐	Unemployed		☐
	- part time	☐	Retired		☐

Q2) What is your age group?
*Please select **one** option only.*

16-24 years	☐	65-74 years	☐
25-34 years	☐	75-84 years	☐
35-44 years	☐	Over 85 years	☐
45-54 years	☐		
55-64 years	☐	Prefer not to say	☐

Q3) On what date was your operation meant to be?
Please provide an estimate if you are unsure of the exact date.

Date: d d - m m - y y y y

Q4) On what date were you told your operation was cancelled or postponed?
Please provide an estimate if you are unsure of the exact date.

Date: d d - m m - y y y y

Q5) If your operation was cancelled or postponed previously, how many times did this occur in total?
Please provide an estimate if you are unsure of the exact number of times.

[ ] times

CODE: [ ]   WES-Pi Study survey Page 1 of 4

Q6) What operation were you meant to be having?
Please describe in your own words, e.g. hernia operation, hip replacement, etc.

Please tell us about the impact of your operation being cancelled or postponed on your work and finances:

Q7) Due to your operation being cancelled or postponed, were you able to **go to work** instead?
Please provide an estimate of the days you think you needed to take off work.

Yes ☐
No* ☐
Not applicable ☐

*If not, how many **days off** did you take?

Q8) Do you think you will need **extra days off work** because your operation was cancelled or postponed?
Please provide an estimate of the day/s you think you will need off work, due to your untreated condition preventing you from working, or the additional time off you will have to take when you receive another date for your operation.

Yes* ☐
No ☐
Not applicable ☐

*If yes, how many **day/s off**?

Q9) Did your friends or family members have to take **extra days off work** because your operation was cancelled or postponed?
Please provide an estimate of the day/s your friends or family members have taken so far, to either look after you when unwell (because you did not have your operation), or planned to take off to look after you following your operation

Yes* ☐
No ☐
Not applicable ☐

*If yes, how many **day/s off**?

emsan.co.uk/wespi wespistudy@gmail.com

Q10) If you are a parent or carer, have you needed **extra childcare** or **carer support**, because your operation was cancelled or postponed?
Please provide an estimate of the total time you have needed so far. e.g. having to book extra support when you are unwell (because you did not have your operation), or needing to rebook extra support for when you receive another operation date.

Yes* ☐
No ☐
Not applicable ☐

*If yes, how many **hours*** or **days***?
****please circle as appropriate***

Q11) Have you had **extra costs in travel and parking** because your operation was cancelled or postponed?
Please provide an estimate if you are unsure of the exact costs so far. e.g. additional journeys to the hospital/doctors because you have been unwell (because you did not have your operation), or because you have needed extra tests before receiving another operation date.

Yes* ☐
No ☐
Not applicable ☐

If yes, how much **extra cost**?

Please tell us about the impact of your operation being cancelled or postponed on your mental health and well-being:

Q12) Are there any key **social activities/hobbies** you have had to miss because your operation was cancelled or postponed?
These may include family days out, attendance at weddings, visiting friends, or your ability to physically participate in your hobbies or interests outside of work.

Yes* ☐
No ☐
Not applicable ☐

*If yes, is there anything specific you would like to mention?

CODE: [ ] WES-Pi Study survey Page 3 of 4

emsan.co.uk/wespi wespistudy@gmail.com

For each question please select **one** option that best describes **your feelings, at the time** your operation was cancelled or postponed:

	Not at all	Slightly	Moderately	Very	Extremely	Not applicable
Q13) How **sad** were you?	☐	☐	☐	☐	☐	☐
Q14) How **disappointed** were you?	☐	☐	☐	☐	☐	☐
Q15) How **angry or annoyed** were you?	☐	☐	☐	☐	☐	☐
Q16) How **frustrated** were you?	☐	☐	☐	☐	☐	☐
Q17) How **stressed** were you?	☐	☐	☐	☐	☐	☐

For each question please select **one** option that best describes **your concerns, at the time** your operation was cancelled or postponed:

	Not at all	Slightly	Moderately	Very	Extremely	Not applicable
Q18) How concerned were you about your **symptoms continuing**?	☐	☐	☐	☐	☐	☐
Q19) How concerned were you about your **health deteriorating**?	☐	☐	☐	☐	☐	☐

Q20) Is there anything else you would like to mention about how your operation being cancelled or postponed has affected you?
e.g. how it made you feel, how it impacted on your life, how it affected you physically, whether you were given enough information, etc.

Thank you for taking the time to complete this survey

Please return it in the pre-paid envelope provided within **two weeks** of receipt.

CODE: [ ] WES-Pi Study survey Page 4 of 4

Table 2: Characteristics of the participants

Characteristic		Number (n)	%
**Age**	<65yrs	176	51.9
	≥65	163	48.1
**Employment status**	Employed	98	28.9
	Self Employed	23	6.8
	Retired	192	56.6
	Unemployed	26	7.7
**Previous cancellation history**	No cancellations	72	25.0
	1 cancellation	106	36.8
	2 cancellations	65	22.6
	≥3 cancellations	45	15.6
**Operations**	General* & Vascular	163	48.1
	Orthopaedic	135	39.8
	Others**	41	12.1
**Returns per hospital**	Nottingham	157	46.3
	Derby	42	12.4
	Chesterfield	42	12.4
	Lincoln	45	13.3
	Leicester	53	15.6

General*: Including general surgery, colorectal, hepatopancreaticobiliary, upper gastrointestinal and endocrine surgery.

Others**: Urology, ENT (ear, nose & throat surgery) and Maxillofacial surgery

For peer review only

Table 3: Responses to questions on psychological impact

	Extremely	Very	Moderately	Slightly	Not at all	Not Applicable
**How sad were you? [n (%)]**	107(32)	56 (17)	45 (13)	70 (21)	40 (12)	21 (6)
**How disappointed were you? [n (%)]**	150 (45)	79 (24)	37 (11)	35 (10)	28 (8)	6 (2)
**How angry were you? [n (%)]**	107 (32)	41 (12)	45 (13)	47 (14)	88 (26)	10 (3)
**How frustrated were you? [n (%)]**	139 (41)	58 (17)	42 (12)	52 (15)	36 (11)	12 (4)
**How stressed were you? [n (%)]**	129 (38)	49 (14)	41 (12)	42 (12)	71 (21)	7 (2)

For peer review only

Table 4: Sample of free text comments from responders

"I was aware of winter pressures and was expecting op to be cancelled. Felt it was right someone with greater need took priority."
"As I am on my own, with no family nearby, it had no real impact. Just a few phone calls to cancel lifts in and back. New appointment for 4th December, so not long to wait. Quite understood situation with the start of the "winter strain", so no point in getting stressed. No complaints at all."
"Struggling to swallow, risk of choking when eating."
"The surgeon was ready to do my operation on the correct day but was told at the last minute there was no bed for me in the ICU/high dependency ward so he couldn't do it. I was very disappointed, but it was not the surgeon's fault."
"I totally understood why my operation was cancelled. My operation needed to be done but my life was not in danger. It was just keeping my work informed so cover could be found whilst I was off work"
"It was cancelled the day before. This meant I had to re-arrange work, so did my partner and the childcare for 2 days. All at late notice. We all work full time. I was also due to fly to America for work and this now became an issue."
"I was disappointed on the original date being cancelled and postponed by 9/10 weeks. It was not a very serious lift threatening situation for me and there were other serious cancer operations that required operations so I fully understood the reasons etc."

For peer review only

"Obviously at the time I felt sad because you not only gear yourself up to have the operation, but I could not justify taking up a hospital bed when there were more needy cases than mine requiring treatment. I do feel more money and less stress for NHS workers is required and the government needs to take heed!"

"I was waiting 6 months for this operation. This meant our lives were "put on hold" for this time. We could not book holidays, arrange anything that involved being away from home as we were expecting op dates; no information on delay times was given. This is very stressful"

"I was surprised to be given pre-med drugs before being told the operation was cancelled. I left the hospital feeling "spaced out"!"

"Each time I came for pre op washout the day before to be told when called in that there is no operation tomorrow. I have a young family so I have to prepare them emotionally."

"I am usually active and my hobbies and enjoyment usually is by way of playing sport. Not being able to undertake these activities had the obvious physical affects and resulted in me being in a low mood, even feeling depressed at times. In turn, I found myself comfort eating and drinking more alcohol. In conclusion, the physical and mental effects were profound."

"Operation performed 5 weeks later, did not affect outcome."

"Cancelled holiday. Developed sepsis after being discharged."

"Very disappointed."

"Such late notice when each operation was cancelled (day before). Very mentally draining preparing for our op then it getting cancelled."

For peer review only

For peer review only - http://bmjopen.bmj.com/site/about/guidelines.xhtml

"I was given a date. When I reached hospital there was no bed. Cancellation happened three times"

"My operation was cancelled due to a trauma case on that day. Everyone at the hospital was so kind and op was arranged for the next week. Thank you."

"My biggest problem has been the effect the original cancellation had on my elderly mother (aged 97) who was booked into respite care for six weeks @ £500 per week. She is still there some six months later. Her chances of coming home have diminished to almost none."

"It was rubbish at the time but completely understandable. Government should take the hit not the NHS. You guys are great and do all you can!"

"I was very distressed, I cried frequently and was very anxious. I felt let down and became depressed. I have not felt like doing anything and have rarely left the house. My irritable bowel syndrome got worse and my mental health has not been good and I have to take medication. My relationships have become strained and I feel I can't trust people."

"It made me feel that I didn't really matter! Although my condition is not life threatening, it is life debilitating."

For peer review only

Appendix 1: PPI in study survey design

No.	Question	PPI Lay Assessors – random comments	Study lead response
Q5	In your own words, what operation were you meant to be having?	Wording change: "Had your operation been previously cancelled or postponed? If so, how many times?"	Amended
Q8	How many **extra** days off work do you now think **you** will miss because of the health condition that you were due to have the operation for?	This is a rhetorical question I doubt even Mr Adiamah could answer correctly.	This has now been amended to reflect the time taken for the surgery and immediately afterwards, and potential time taken off due to ongoing illness related to the cancellation
Q10	How much extra cost has **childcare** cost you because of your operation being cancelled or postponed?	Question on childcare costs. May not be relevant to all participants, and potentially quite intrusive. Also, costs can be very variable.<br><br>Suggestion of using 'hours of childcare' instead.	Question amended to ask about 'hours of childcare<br><br>AND carer support' if relevant.<br><br>Descriptor added.
Q13-Q18	Please answer the following questions in relation to your operation being cancelled or postponed.<br><br>*For each question please select ONE option that best describes your feelings* ***today.***<br><br>How **disappointed (Q13); angry (Q14); frustrated (Q15); stressed (Q16)** are you about your operation?<br><br>How concerned are you about	Surely 'today' might not be a good measure of feelings about the change in plan	Amended to ask about feelings at the time of the postponement instead of 'today'.

For peer review only - http://bmjopen.bmj.com/site/about/guidelines.xhtml

	your **symptoms continuing**? (Q17); **health deteriorating**? (Q18)		
Overall	Additional box for other comments (this was suggested by two of the PPI Lay Assessors)		Great suggestion, thanks – we have added this.

For peer review only

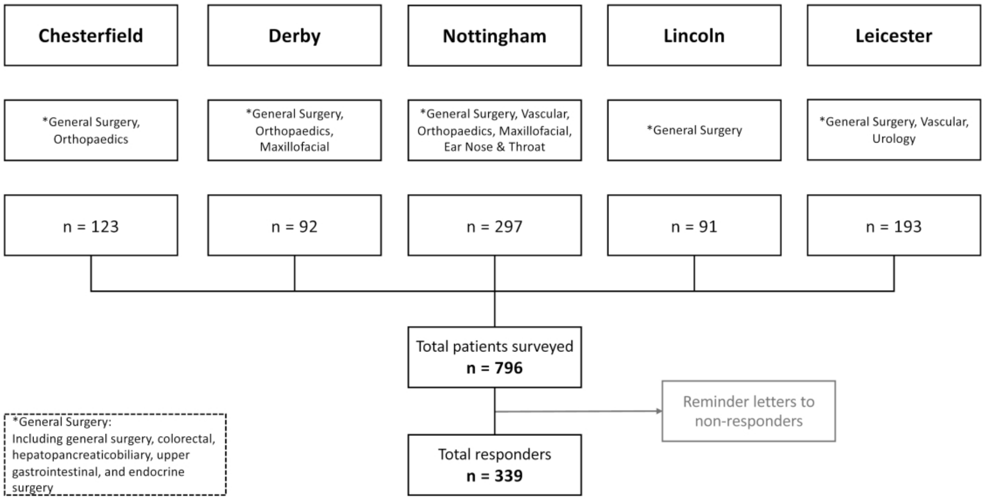
Chesterfield
Derby
Nottingham
Lincoln
Leicester
*General Surgery, Orthopaedics
*General Surgery, Orthopaedics, Maxillofacial
*General Surgery, Vascular, Orthopaedics, Maxillofacial, Ear Nose & Throat
*General Surgery
*General Surgery, Vascular, Urology
n = 123
n = 92
n = 297
n = 91
n = 193
Total patients surveyed n = 796
Reminder letters to non-responders
Total responders n = 339
*General Surgery: Including general surgery, colorectal, hepatopancreaticobiliary, upper gastrointestinal, and endocrine surgery

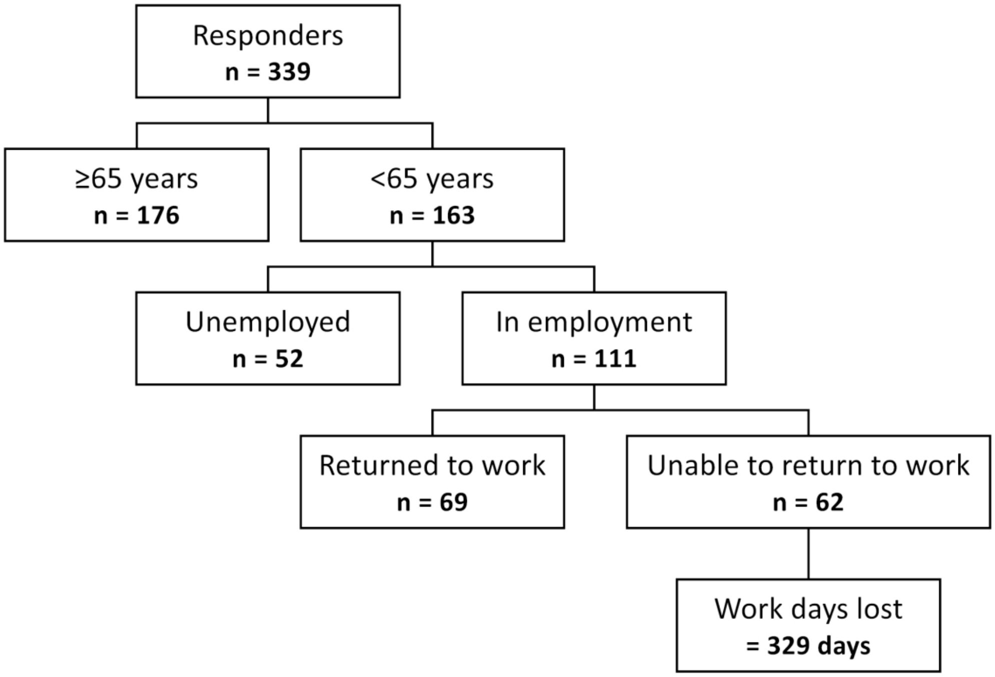
Responders
n = 339
≥65 years
n = 176
<65 years
n = 163
Unemployed
n = 52
In employment
n = 111
Returned to work
n = 69
Unable to return to work
n = 62
Work days lost
= 329 days

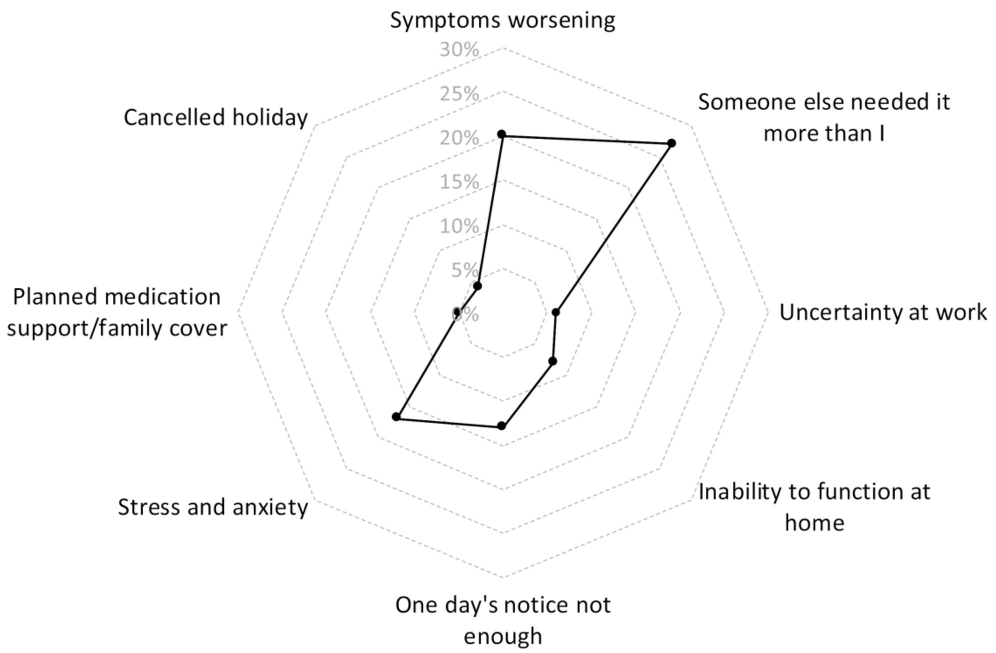
Symptoms worsening
Someone else needed it more than I
Uncertainty at work
Inability to function at home
One day's notice not enough
Stress and anxiety
Planned medication support/family cover
Cancelled holiday
30%
25%
20%
15%
10%
5%
0%

National Institute for Health Research

WES-Pi

1st March 2018

Dear Patient,

As you may be aware, due to the increased pressures faced by the NHS during the winter period, some patients like yourself have unfortunately had their operations either cancelled or delayed.

To understand the true impact this can have on our patients' lives, we are asking all patients who have experienced a cancellation or delay to complete the attached short survey (Winter Elective Surgery Cancellation and Psychological Impact survey).

You do not have to participate in this survey, but your experiences may help us to improve our services for future patients. There are no right or wrong answers to any of the questions asked, it is your opinions that we are really interested in. In particular, we would like to hear how your experience of a cancellation has affected you emotionally, physically, and financially.

You do not have to give any personal details, and the results of the survey will be completely anonymous when published. To participate, please fill in the attached survey and return it to us in the pre-paid envelope provided. If you have any further questions about this survey you can contact us at: wespistudy@gmail.com

Thank you for your time.

Yours sincerely,

The East Midlands Surgical Academic Network (EMSAN)

EMSAN is a group of trainee doctors who work for the NHS. We believe in making research opportunities available to everyone, to aid learning and improve medical services.

Visit us at: emsan.co.uk/wespi
Contact us at: wespistudy@gmail.com

For peer review only

BMJ Open

Winter cancellations of elective surgical procedures in the United Kingdom: A questionnaire survey of patients on the economic and psychological impact

Journal:	*BMJ Open*
Manuscript ID	bmjopen-2018-028753.R1
Article Type:	Research
Date Submitted by the Author:	07-Jun-2019
Complete List of Authors:	Herrod, Philip; Royal Derby Hospital, Surgery<br>Adiamah, Alfred; University of Nottingham<br>Boyd-Carson, Hannah; Royal Derby Hospital<br>Daliya, Prita; University of Nottingham<br>El-Sharkawy, Ahmed; Royal Derby Hospital<br>Sarmah, Panchali; University Hospitals of Leicester NHS Trust<br>Hossain, Tanvir; Nottingham University Hospitals NHS Trust<br>Couch, Jennifer; Nottingham University Hospitals NHS Trust, Department of General Surgery<br>Sian, Tanvir; Royal Derby Hospital<br>Wragg, Andrew; University of Nottingham, Nottingham Digestive Diseases Centre<br>Andrew, David; Lincoln County Hospital<br>Parsons, Simon; Nottingham University Hospitals NHS Trust, Department of General Surgery<br>Lobo, Dileep; University of Nottingham, Nottingham Digestive Diseases Centre
**Primary Subject Heading**:	Surgery
Secondary Subject Heading:	Qualitative research, Patient-centred medicine, Medical management
Keywords:	SURGERY, Adult surgery < SURGERY, QUALITATIVE RESEARCH, Organisation of health services < HEALTH SERVICES ADMINISTRATION & MANAGEMENT

SCHOLARONE™
Manuscripts

Winter cancellations of elective surgical procedures in the United Kingdom: A questionnaire survey of patients on the economic and psychological impact

Philip J J Herrod*[1,2], Alfred Adiamah*[1,2], Hannah Boyd-Carson[1,2], Prita Daliya[1,2], Ahmed M El-Sharkawy[1,2], Panchali B Sarmah[1,3], Tanvir Hossain[1,2], Jennifer Couch[1,2], Tanvir S Sian[1,2], Andrew Wragg[4], David R Andrew[5], Simon L Parsons[1,2], Dileep N Lobo[1,2,6], WES-Pi Study Group on behalf of the East Midlands Surgical Academic Network (EMSAN)†

*Joint first authors

[1]East Midlands Surgical Academic Network, Queen's Medical Centre, NG7 2UH, UK
[2]Gastrointestinal Surgery, Nottingham Digestive Diseases Centre and National Institute for Health Research (NIHR) Nottingham Biomedical Research Centre, Nottingham University Hospitals and University of Nottingham, Queen's Medical Centre, Nottingham NG7 2UH, UK
[3]Leicester Cancer Research Centre, University of Leicester, LE2 7LX, UK
[4]Patient & Public Involvement Facilitator, Gastrointestinal and Liver Disorders Theme, National Institute for Health Research (NIHR) Nottingham Biomedical Research Centre, Nottingham University Hospitals NHS Trust and the University of Nottingham, Queen's Medical Centre, Nottingham NG7 2UH, UK
[5]Lincoln County Hospital, Greetwell Road, Lincoln LN2 5QY, UK
[6]MRC/ARUK Centre for Musculoskeletal Ageing Research, School of Life Sciences, University of Nottingham, Queen's Medical Centre, Nottingham NG7 2UK, UK

† WES-Pi Study Group:

Jennifer Couch, Prita Daliya, Tanvir Hossain, Bethan Johnson, Amanda Koh, Anisa Kushairi, Dileep N Lobo, Simon L Parsons, Andrew Wragg (Nottingham University Hospitals NHS Trust); Alfred Adiamah, David R Andrew, Chris Lewis-Lloyd, Farah Roslan, Amari Thompson, Helen Wan (United Lincolnshire Hospitals NHS Trust); Ahmed M El-Sharkawy, Sita Kotecha, Kevin Sargen (Chesterfield Royal Hospitals NHS Foundation Trust); Hannah Boyd-Carson, Phillip J J Herrod, Jonathan N Lund, Alastair Morton, Tanvir S Sian (Derby Teaching Hospitals NHS Foundation Trust); Edmund Charles, Panchali B Sarmah, Baljit Singh (University Hospitals of Leicester NHS Trust).

Address for correspondence:
Professor Dileep N Lobo
Gastrointestinal Surgery
Nottingham Digestive Diseases Centre
Nottingham University Hospitals NHS Trust and University of Nottingham
E Floor, West Block
Queen's Medical Centre
Nottingham NG7 2UH, UK
Tel: +44-115-8231149
Fax: +44-115-8231160
E-mail: Dileep.Lobo@nottingham.ac.uk

For peer review only

Category: Original research

Word count (excluding title page, abstract, figure legends, tables and references): 2961

Table and figures: 3 tables and 3 figures + 1 supplementary digital document

Running head: Winter Elective Surgery Cancellation and Psychological Impact (WES-Pi) Survey

Key words: winter cancellations; elective operations; psychosocial impact; economic impact; National Health Service

Funding: This work was supported by the Medical Research Council [grant number MR/K00414X/1]; and Arthritis Research UK [grant number 19891]. AA was funded by a National Institute for Health Research (NIHR) Academic Clinical Fellowship.

The funders had no role in the design or conduct of the work, or in the decision to publish. This paper presents independent research funded by the MRC, ARUK and the NIHR. The views expressed are those of the authors and not necessarily those of the MRC, ARUK, NHS, the NIHR or the Department of Health.

Conflict of interest: None of the authors has a direct conflict of interest to declare. SLP is a company director for EIDO Healthcare Limited (unrelated to the submission). DNL has received an unrestricted research grants for unrelated work from BBraun in the last 3 years. He has also received speakers' honoraria from BBraun, Fresenius Kabi, Shire and Baxter Healthcare for unrelated work in the last 3 years.

This paper was to the Annual Meeting of the Society for Academic and Research Surgery in London, UK on 8 January 2019. It will be published in abstract form in the *British Journal of Surgery*.

"The Corresponding Author has the right to grant on behalf of all authors and does grant on behalf of all authors, a worldwide licence to the Publishers and its licensees in perpetuity, in all forms, formats and media (whether known now or created in the future), to i) publish, reproduce, distribute, display and store the Contribution, ii) translate the Contribution into other languages, create adaptations, reprints, include within collections and create summaries, extracts and/or, abstracts of the Contribution, iii) create any other derivative work(s) based on the Contribution, iv) to exploit all subsidiary rights in the Contribution, v) the inclusion of electronic links from the Contribution to third party material where-ever it may be located; and, vi) licence any third party to do any or all of the above."

For peer review only

ABSTRACT

Objectives: To quantify the economic and psychological impact of the cancellation of operations due to winter pressures on patients, their families and the economy.

Design: This questionnaire study was designed with the help of patient groups. Data were collected on the economic and financial burden of cancellations. Emotions were also quantified on a 5-point Likert scale.

Setting: Five NHS Hospital Trusts in the East Midlands region of England.

Participants: We identified 796 participants who had their elective operations cancelled between 1-11-2017 and 31-3-2018 and received responses from 339 (43%).

Interventions: Participants were posted a modified version of a validated quality of life questionnaire with a pre-paid return envelope.

Main Outcome Measures: The primary outcome measures were the financial and psychological impact of the cancellation of elective surgery on patients and their families.

Results: Of the 349 respondents, 163 (48%) were aged <65 years, with 111 (68%) being in employment. Sixty-six (19%) had their operations cancelled on the day. Only 69 (62%) of working adults were able to return to work during the time scheduled for their operation, with a mean (standard deviation, SD) loss of 5 working days (SD 10). Additional working days were lost subsequently by 60 (54%) participants [mean 7 days (SD 10)]. Family members of 111 (33%) patients required additional time off work [mean 5 days (SD 7)]. Over 30% of participants reported extreme levels of sadness, disappointment, anger, frustration and stress. At least moderate concern about continued symptoms was reported by 234 (70%)

For peer review only

participants, and 193 (59%) reported at least moderate concern about their condition deteriorating.

Conclusions: The cancellation of elective surgery during the winter had an adverse impact on patients and the economy, including days of work lost and health-related anxiety. We recommend better planning, and provision of more notice and better support to patients.

For peer review only

Strengths

- This study presents aggregated evidence to support previous anecdotes of the importance of cancellation of elective operations to patients' well-being. It was devised with active consultation with patient groups. The questions asked were those that the patient groups thought relevant to the problem.
- It highlights the importance of early notification of cancellations in times such as winter pressures, where cancellations are unavoidable to sustain the delivery of emergency care.

Limitations

- This study was retrospective by design and was potentially limited by recall bias
- We achieved a less than 50% responder rate, in spite of two rounds of questionnaires being sent, and as such is subject to non-responder bias.
- The study covered a single but large region of the United Kingdom, it is difficult to ascertain if the population dynamics and level of social deprivation make this population similar to those in all parts of the UK, and as such affects the generalisability of our results.

For peer review only

INTRODUCTION

The winter of 2017-2018 saw an unsustainable increase in patient demand on acute hospital care in England, due both to an anticipated seasonal rise in admissions and an unexpectedly severe epidemic of winter flu. In response to what was dubbed the "Winter National Health Service (NHS) Crisis" by the media, NHS England took the unprecedented step of instructing all NHS hospital trusts to cancel planned routine non-cancer surgery throughout December 2017 and January 2018, in order to free resources for emergency admissions.[1]

Although official figures have not yet been released, media estimates put the number of patients affected in England at around 50,000.[2] This huge volume of cancellations will have ongoing ramifications for the 2018 calendar year. The rescheduling of a large backlog of cancelled operations by hospital trusts, will undoubtedly put added pressure on hospital waiting lists.

Despite the effect of a cancellation on the ongoing care provided by hospitals being subject to many reporting structures, the psychological and economic impact of a cancellation on patients themselves has received remarkably little attention. The requirement of a period of recuperation, means most patients in their preparation for elective surgery make arrangements in their social lives to facilitate this. Adults in employment may book time off work and postpone important social events such as holidays. Carers may similarly organise additional support or childcare arrangements. Family members may also do the same in order to help care for recovering patients. If a planned operation is postponed at short notice, it is possible to envisage these arrangements resulting in personal and economic loss.

For peer review only

Any rescheduled operative date may also result in patients and carers incurring the same costs again with no recourse to reimbursement. This may lead to a considerable economic burden on the patient and their family[3] and cause additional loss of working days for the economy. Postponement or cancellation of elective surgery may also have a profound psychological impact on patients and their families, who may have mentally prepared themselves for the event. There may also be significant health-related anxiety associated with a longer wait for surgery, as patients may fear their condition deteriorating further.[4] We aimed, in this **W**inter **E**lective **S**urgery Cancellation and **P**sychological **i**mpact (WES-Pi) survey, to quantify the financial and psychological impact on patients following the postponement or cancellation of elective surgery in England, due to winter pressures.

For peer review only

METHODS

This multicentre study was conducted across five NHS acute hospital trusts (Nottingham University Hospitals NHS Trust, University Hospitals of Leicester NHS Trust, Derby Teaching Hospitals NHS Foundation Trust, United Lincolnshire Hospitals NHS Trust and Chesterfield Royal Hospitals NHS Foundation Trust)in the East Midlands region of England during the winter of 2017-18 in accordance with the STROBE statement.[5] Adult patients aged 16 years or over, scheduled for elective surgery between 1 November 2017 and 31 March 2018 were identified from local hospital waiting list records. Participants were chosen from the surgical subspecialties of general surgery, colorectal, hepatopancreaticobiliary, upper gastrointestinal, endocrine, vascular, urology, orthopaedics, maxillofacial, and ear nose and throat. All patients who had their elective surgery postponed or cancelled for non-patient related reasons were included (**Figure 1**). Patients who had surgery postponed or cancelled for patient-related reasons (e.g. unrelated illness, medically unfit, or surgery declined), or those that died before dispatch of the questionnaires were not invited to participate.

Outcomes

Our primary outcome measure was to quantify the financial and psychological impact of cancellation of elective surgery on participants and their families. The secondary outcome measure was to evaluate the impact of the length of notice of cancellation on the primary outcomes.

For peer review only - http://bmjopen.bmj.com/site/about/guidelines.xhtml

Survey design

In the absence of an existing validated questionnaire to explore financial or emotional consequences to a patient, specifically related to the cancellation of elective surgery, we modified a previously used survey from a paediatric study.[6] Survey design involved multiple iterations and amendments through patient focus groups and significant involvement and input from the Patient Advisory Group at Nottingham University Hospitals NHS trust, and the Patient Advisory and Liaison Service (PALS) team at Derby Teaching Hospitals NHS Trust. In addition to gathering data on the economic and financial burden of postponements and cancellations on participants and their relatives, the questionnaire also quantified emotional feelings of participants on a 5-point Likert scale[7] (**Appendix 1**).

Patient and public involvement

Patient and Public Involvement (PPI) was an integral element throughout this study given its direct relevance to patient care. Embedding PPI into to the study from the outset meant we could more accurately explore the patient experience of cancellations informed by what patients felt from prior experience were important in their care. There is a current drive for PPI in all medical research, for experiential reasons (Patients or carers have experience of a condition, treatment or in this instance experience of a cancelled appointment for elective operation); ethical and moral reasons (Patients and the public are directly affected by the research process and findings; they have the right to contribute to it; and of course are the tax payers); and pragmatic reasons (PPI is a requirement for funding bodies; it translates

For peer review only

research into knowledge). In this instance, we felt strongly that for a study where the purpose was solely about patient experience, it had to be informed fully by patients.

We chose to recruit PPI Lay Assessors to review the invitation letter and subsequent survey to be sent to study participants. We wanted to prove that the intent to use a survey to explore the impact of cancelled elective operations on the psychosocial wellbeing of our patients, as a means leading to service improvement, if suitably structured would achieve its outcomes. PPI provided assurance that the devised series of questions contained in the survey was suitable to cover the aspects of financial, psychological and physical impact of these cancellations including symptomatic (suggestive) deterioration without adversely impacting on patients or being too onerous to complete.

They directed the changes through several iterations and approved of the final survey and invitation letter, ensuring the overall suitability and acceptability of the survey.

How was the development of the research question and outcome measures informed by patients' priorities, experience, and preferences?

In consultation with PPI members, the research tool was shaped to more accurately assess the financial impact of cancellations, not only for patients but also for their family members involved in their care. In exploring patients' emotions, their responses allowed us to make the questions more specific to feelings at the time of cancellations (**Appendix 1**)

How did you involve patients in the design of this study?

For peer review only - http://bmjopen.bmj.com/site/about/guidelines.xhtml

From the PPI perspectives, the measures were informed via six (6) recruited PPI members from the Gastrointestinal & Liver Patient Advisory Group, charged with reviewing these study public-facing documents (invitation letter and survey to be issued to the study participants). Each lay PPI Member assessed and reported back their respective thoughts on the document(s) content, layout and its message. Examples of comments (on the survey) made, and responses made by study leads are presented in **Appendix 2**

Were patients involved in the recruitment to and conduct of the study?

No. Recruitment was informed by the cancellation list at each participating trust.

How will the results be disseminated to study participants?

The write up of the manuscript included the lead person from the Gastrointestinal and Liver PPI group, allowing the patient viewpoints to be more accurately reflected in our results. It also ensures dissemination to the initial group who were involved in the study design. More importantly there is an opportunity for this study to aid advocacy in the conduct of patient cancellations in a manner that results in the least adverse outcomes for patients.

For randomised controlled trials, was the burden of the intervention assessed by patients themselves?

Not applicable.

Ethics and Data collection

For peer review only - http://bmjopen.bmj.com/site/about/guidelines.xhtml

The study was registered and approved locally at each participating hospital trust, under the supervision of a named consultant surgeon as a service improvement project. The project registration/approval numbers for each site were: Nottingham University Hospitals NHS Trust 18-050c, University Hospitals of Leicester NHS Trust 9339E, Derby Teaching Hospitals NHS Foundation Trust SB-Gen-2017/18 -1025, United Lincolnshire Hospitals NHS Trust 1301 and Chesterfield Royal Hospitals NHS Foundation Trust WES-Pi 001. Identified participants were posted a copy of the study questionnaire (**Appendix 1**), a pre-paid return envelope, and an invitation letter (**Appendix 2**), incorporating local trust branding. Invitations were first posted to participants in April 2018, with a further invitation to non-responders in June 2018. Each study site was managed by local teams responsible for local study registration and approvals, identification of participants, dissemination of postal invitations, and data collection. Returned surveys were pseudoanonymised, with collated data subsequently anonymised and encrypted before central data analysis. No identifiable participant data were transferred between sites.

Data synthesis

Quantitative and qualitative data analyses of responses were undertaken. Quantitative data analysis was performed using STATA® v15.0 (StataCorp, College Station, TX, USA). Linear regression was performed to assess for correlation and a chi-square test to assess for differences between categorical data. Qualitative data was evaluated using NVivo v11 (QSR International Inc., Burlington, MA, USA) for thematic analysis. All descriptive data are represented as n (%), mean (standard deviation, SD) or median (inter quartile range, IQR) as appropriate.

For peer review only

RESULTS

Return rate and cancellation

Questionnaires were posted to 796 participants and returned by 339 (43%) after two distribution rounds. The response rate varied between trusts from 27% to 52%. Data on participant characteristics and surgical specialties involved are displayed in **Table 1**.

Of the participants returning questionnaires, 66 (19%) had their operations cancelled on the day of surgery, 44% (n=149) within 24 hours of the proposed surgery, and 37% (n=125) cancelled more than 48 hours from the proposed date of the operation. Median notification of cancellation was 2 days (IQR 1-6). A number of participants had been cancelled previously prior to the winter cancellation. The responders reported their operation as having been cancelled a median of 1 time (IQR 1-2) previously. In 15% (45/288) of the responders, operations had been cancelled three times or more (**Table 1**).

Economic burden

One hundred and sixty-three (48%) of responding participants were aged less than 65 years and of these 111 (68%) were in employment. Only 69 (62%) of the working adults were able to return to work during the time they had originally scheduled off for their operation, with a median loss of 1 day (IQR 0-5) of work. The total number of work days lost in the cohort was 329 (**Figure 2**).

Additional working days in subsequent months were lost by 60 (54%) of working adults due to the cancellation [median 3 days (IQR 0-12)], totalling 456 days. Family members of 111

For peer review only - http://bmjopen.bmj.com/site/about/guidelines.xhtml

(33%) participants required additional time off work median 3 days (IQR 1-5), totalling 581 days. Thus, the total number of missed days of work (from participants and family members, both from the initial cancellation and subsequent rescheduling of the operation) was 1366 days. There was no direct correlation between days of work lost and the notification given for cancellation (r^2=0.0004, p=0.87).

Child care costs and travel costs

Of the responders with children, 23 were forced to organise extra childcare. Of these the median (IQR) additional childcare days required was 4 days (0-22.5). One hundred and twenty-three participants (48%) incurred additional travel costs following cancellation of their operation. The median cost incurred for additional travel was £15 (IQR £6.5-£30).

Psychological burden

The majority of participants reported at least moderate levels of sadness, disappointment, anger, frustration and stress, with over 30% (n=102) of respondents reporting extreme levels of each of these emotions and over 50% of respondents reporting at least “very” (the second highest answer available) to each of these emotions. A full breakdown of emotions reported is displayed in **Table 2**. There was no significant difference in the reporting of these emotions between patients who had been cancelled only once or those who had suffered multiple cancellations. At least moderate concern about continued symptoms was reported by 234

For peer review only - http://bmjopen.bmj.com/site/about/guidelines.xhtml

(70%) patients whilst waiting for a rescheduled date whilst 193 (59%) reported at least moderate concern about their condition deteriorating.

There was no correlation between strength of any emotion and the amount of notice given to participants for the cancellation. This was true for all the emotive questions asked.

Thematic analysis from free text responses

The qualitative analysis of the participants' free text responses is presented in the thematic analysis as a radar diagram (**Figure 3**). Four particular themes were highlighted in this analysis. There was concern over symptoms worsening; stress and anxiety related to the cancellation; and those whose operations were cancelled on the day felt that this was unsatisfactory and caused them undue distress. However, the most interesting finding on the free text analysis was that 26% (70/268) of the responders understood the need for their operations to be cancelled to facilitate delivery of the emergency service. They used such descriptions as: "my operation was not life-threatening" and "others needed the care more" (**Table 3**).

For peer review only

DISCUSSION

The uncharacteristically cold weather, the high rates of emergency admissions, particularly in the elderly, and a lack of capacity in an already overworked and overstretched system gave rise to the perfect storm of cancellations during the winter of Nov 2017 to Feb 2018. Some even referred to it as another winter of discontent for the NHS.[8] The national strategy, instigated by NHS England was to cancel elective operations to release capacity in the system.[1] These elective cancellations were well publicised in the media, and meant that the majority of the population had some idea of the unusual stress the NHS was under.[2 8] However, despite public awareness, the impact on patients of these cancellations and, in particular last minute cancellations, was significant.

What this study found

This study is the first in England to evaluate the impact of the mass cancellation of elective surgery on patients, from both an economic and psychological view point. We have demonstrated that a large proportion of patients suffered a negative economic impact from both the additional work days lost and the additional non-refundable travel and childcare costs. We achieved this using a novel research tool, developed jointly with patient groups to establish usability, acceptability and internal validity.

Taken in the context of a nation-wide bed pressure crisis, which was widely publicised in the media, the fact that nearly 20% of patients had their operations cancelled on the day of surgery was surprising. Despite this we were unable to demonstrate any correlation between the period of notice given for cancellation of the planned operation, and the number of working days lost, nor any impact on the responses that patients gave to the

For peer review only

questions on their emotional state. Although the reasons behind this are unclear, our study may have been underpowered to demonstrate this and also due to the over representation of retired patients in our cohort.

Our relatively small sample from one region of the UK, comprising 5 hospitals, contained nearly 800 cancelled operations and in those responders led to the loss of 1366 work days to the economy from our modest 43% response rate. Given that this crisis stretched to the whole length of the UK and was estimated to have led to 50,000 cancellations nationwide, the projected loss of working days may exceed 85,000 days, even on the unlikely assumption that the non-responding patients lost no days from work. The questions on psychological impact, demonstrated that elective surgical cancellation has led to a large amount of negative emotion amongst patients, with the majority displaying at least a moderate amount of health-related anxiety.

Although this study was about winter cancellations of operations of patients from one region of England, the results are probably generalisable to the rest of the UK and also to other countries. Moreover, the results are not just applicable to winter cancellations, but could be applied to cancellations occurring at other times of the year as well.

What is available in the literature?

Whilst several studies have evaluated the causes of bed pressures and failures of hospital trusts to meet the emergency department 4-hour performance target[9 10] and others have evaluated the reason for elective cancellations across a range of health care settings,[11 12] very little information is available on the psychosocial impact of cancellations of elective operations on patients. Others have reported on measures to reduce same day cancellations

For peer review only

of elective operations, but have focused on other aspects, such as ensuring fitness for surgery, preventing the scheduling of the wrong operation, or preventing incomplete preoperative assessments rather than addressing cancellations due to a lack of beds.[13] Others have counted the cost of such last minute cancellations to the hospital itself, from specialised equipment that might need to be brought in, and noted that cancellations within 24 hours impacted significantly on costs to the trust.[14] In this study we found that 19% of the patients had their operations cancelled within 24 hours. Whilst this resulted in a financial impact on the patients, such late cancellations are also likely to increase costs for the hospital trust itself. From the point of view of optimal deployment of personnel and resources, better planning is needed for future crises. The Royal College of Surgeons of England has published guidance to help manage winter pressures and has acknowledged that it was better for patients to be told in advance of their cancellations in agreement with the findings of this study.[15] Additionally, they acknowledged the consequences of such cancellations inevitably would include prolonged suffering of the patients affected.[15] Better forecast and long-term planning of population health needs by policy makers are the only ways to avert these winter cancellations from becoming a recurring theme in the delivery of healthcare in the UK.

Limitations

Our study may be limited by its response rate, potential recall bias and non-responder bias. We took steps to maximise our response rate by sending pre-paid return envelopes with each questionnaire, and by dispatching a second round of questionnaires to non-responders. However, despite these efforts we were unable to improve our response rate above 50%. We would contend, however, that even with the unlikely assumption that the non-

For peer review only

responders did not experience any negative emotion or loss of work, the economic and psychological burden on this cohort was considerable.

CONCLUSIONS

The WES-Pi survey has provided evidence to support the often-cited anecdotes concerning the impact of cancelling elective operations on patients' finances and psychological wellbeing. The national awareness of cancellations meant that the majority of responding patients understood the need for these cancellations. However, with 20% of respondents having their operations cancelled on the day of the procedure better planning is needed in the future to reduce cancellations and to provide more notice and better support to patients who have their operations cancelled. This may require a national change in policy by the Department of Health, in collaboration with specialist clinical commissioning groups and local trusts.

For peer review only

REFERENCES

1. Campbell D, Duncan P. NHS cancels surgery for tens of thousands to avoid winter crisis. *The Guardian* 21 December, 2017. Available at https://www.theguardian.com/society/2017/dec/21/nhs-cancels-surgery-tens-of-thousands-avoid-winter-crisis Accessed 10 November 2018.
2. Donnelly L, Bodkin H. NHS hospitals ordered to cancel all routine operations in January as flu spike and bed shortages lead to A&E crisis *The Telegraph* 2 January 2018 Available at https://www.telegraph.co.uk/news/2018/01/02/nhs-hospitals-ordered-cancel-routine-operations-january/ Accessed 10 November 2018.
3. Lankoandé M, Bonkoungou P, Ki BK, et al. Economic and psychological burden of scheduled surgery cancellation in a sub-Saharan country (Burkina Faso). Southern African Journal of Anaesthesia and Analgesia 2017;**23**(6):145-51.
4. Carr T, Teucher U, Mann J, et al. Waiting for surgery from the patient perspective. Psychol Res Behav Manag 2009;**2**:107-19.
5. von Elm E, Altman DG, Egger M, et al. Strengthening the Reporting of Observational Studies in Epidemiology (STROBE) statement: guidelines for reporting observational studies. BMJ 2007;**335**(7624):806-8.
6. Tait AR, Voepel-Lewis T, Munro HM, et al. Cancellation of pediatric outpatient surgery: economic and emotional implications for patients and their families. J Clin Anesth 1997;**9**(3):213-9.
7. Sullivan GM, Artino AR, Jr. Analyzing and interpreting data from likert-type scales. J Grad Med Educ 2013;**5**(4):541-2.
8. Campbell G. WINTER OF DISCONTENT? Plan to avoid hospital crisis at Christmas as pressure on A&E increases. *Oxford Mail* 17 November 2017. Available at https://www.oxfordmail.co.uk/news/15666401.winter-of-discontent-plan-to-avoid-hospital-crisis-at-christmas-as-pressure-on-ae-increases/ Accessed 10 November 2018
9. Keogh B, Culliford D, Guerrero-Luduena R, et al. Exploring emergency department 4-hour target performance and cancelled elective operations: a regression analysis of routinely collected and openly reported NHS trust data. BMJ Open 2018;**8**(5):e020296.
10. Morris AJ, McAvoy J, Dweik D, et al. Cancellation of elective cases in a recently opened, tertiary/quaternary-level hospital in the Middle East. Anesth Analg 2017;**125**(1):268-71.
11. Desta M, Manaye A, Tefera A, et al. Incidence and causes of cancellations of elective operation on the intended day of surgery at a tertiary referral academic medical center in Ethiopia. Patient Saf Surg 2018;**12**:25.
12. Kumar R, Gandhi R. Reasons for cancellation of operation on the day of intended surgery in a multidisciplinary 500 bedded hospital. J Anaesthesiol Clin Pharmacol 2012;**28**(1):66-9.
13. McKendrick DR, Cumming GP, Lee AJ. A 5-year observational study of cancellations in the operating room: Does the introduction of preoperative preparation have an impact? Saudi J Anaesth 2014;**8**(Suppl 1):S8-S14.

For peer review only

14. Sanjay P, Dodds A, Sanjay P, Miller E, Arumugam PJ, Woodward A. Cancelled elective operations: an observational study from a district general hospital. J Health Organ Manag 2007;**21**(1):54-58.
15. Royal College of Surgeons of England. Guidance to help manage winter pressures. 28 November 2018. Available at https://www.rcseng.ac.uk/news-and-events/news/archive/guidance-to-help-manage-winter-pressures/ Accessed 12 December 2018.

For peer review only

Statements

Competing interest statement

All authors have completed the Unified Competing Interest form at http://www.icmje.org/coi_disclosure.pdf (available on request from the corresponding author) and declare: no support from any organisation for the submitted work; no financial relationships with any organisations that might have an interest in the submitted work in the previous three years, no other relationships or activities that could appear to have influenced the submitted work." Please see http://resources.bmj.com/bmj/authors/editorial-policies/competing-interests

Exclusive licence

The Corresponding Author has the right to grant on behalf of all authors and does grant on behalf of all authors, an exclusive licence (or non exclusive for government employees) on a worldwide basis to the BMJ Publishing Group Ltd and its Licensees to permit this article (if accepted) to be published in BMJ editions and any other BMJPGL products and sublicences to exploit all subsidiary rights, as set out in our licence)."

Contributorship statement

Study design: PJJH, AA, HBC, PD, AME-S, PBS, TH, JC, TSS, DRA, AW, SLP, DNL

Development of questionnaire: AA, PJJH, PD, HBC, AW

Data collection: PJJH, AA, HBC, PD, AME-S, PBS, TH, JC, TSS, DRA, AW, SLP, DNL

Data-analysis: PJJH, AA, HBC

Data-interpretation: PJJH, AA, HBC, PD, DNL

Writing of Manuscript: PJJH, AA, PD, HBC, DNL

Creation of figures: AA, PD, DNL

Critical review of Manuscript: DRA, AW, SLP, DNL

Final approval: PJJH, AA, HBC, PD, AME-S, PBS, TH, JC, TSS, DRA, AW, SLP, DNL

PPI member's acknowledgements

We are very grateful to the following PPI members from the Gastrointestinal & Liver Patient Advisory Group, who worked on the questionnaire tool and whose contributions overall

For peer review only

shaped the study design. They are: Karen Andrews; David Harrison; David Matthews; John Shaw; Sally Taylor; and Stevie Vanhegan.

Guarantor Statement

The lead author (the manuscript's guarantor) affirms that the manuscript is an honest, accurate, and transparent account of the study being reported; that no important aspects of the study have been omitted; and that any discrepancies from the study as planned (and, if relevant, registered) have been explained.

Ethical approval

The study was registered and approved locally at each participating hospital trust, under the supervision of a named consultant surgeon as a service improvement project. The project registration/approval numbers for each site were: Nottingham University Hospitals NHS Trust 18-050c, University Hospitals of Leicester NHS Trust 9339E, Derby Teaching Hospitals NHS Foundation Trust SB-Gen-2017/18 -1025, United Lincolnshire Hospitals NHS Trust 1301 and Chesterfield Royal Hospitals NHS Foundation Trust WES-Pi 001.

Data sharing

Data will be available upon reasonable request from Mr. Philip J J Herrod (pherrod@nhs.net).

Patient involvement

The survey design involved multiple iterations and amendments through patient focus groups and significant involvement and input from the Patient Advisory Group at Nottingham University Hospitals NHS trust, and the Patient Advisory and Liaison Service (PALS) team at Derby Teaching Hospitals NHS Trust. One of the authors (AW) is a Patient & Public Involvement Facilitator.

For peer review only

LEGENDS FOR FIGURES

Figure 1: Study flow diagram.

Figure 2: Number of work days lost.

Figure 3: Radar diagram of thematic analysis (figures represent percentage of responders).

For peer review only

Table 1: Characteristics of the participants

Characteristic		Number (n)	%
**Age**	<65yrs	176	51.9
	≥65	163	48.1
**Employment status**	Employed	98	28.9
	Self Employed	23	6.8
	Retired	192	56.6
	Unemployed	26	7.7
**Previous cancellation history**	No cancellations	72	25.0
	1 cancellation	106	36.8
	2 cancellations	65	22.6
	≥3 cancellations	45	15.6
**Operations**	General* & Vascular	163	48.1
	Orthopaedic	135	39.8
	Others**	41	12.1
**Returns per hospital**	Nottingham	157	46.3
	Derby	42	12.4
	Chesterfield	42	12.4
	Lincoln	45	13.3
	Leicester	53	15.6

General*: Including general surgery, colorectal, hepatopancreaticobiliary, upper gastrointestinal and endocrine surgery.

Others**: Urology, ENT (ear, nose & throat surgery) and Maxillofacial surgery

For peer review only

Table 2: Responses to questions on psychological impact

	Extremely	Very	Moderately	Slightly	Not at all	Not Applicable	P
**How sad were you? [Total n (%)]**	107 (32)	56 (17)	45 (13)	70 (21)	40 (12)	21 (6)	
1 cancellation **[n (%)]**	54 (30)	27 (15)	28 (15)	41 (23)	21 (12)	10 (6)	
>1 cancellation **[n (%)]**	53 (34)	29 (18)	17 (11)	29 (18)	19 (12)	11 (7)	0.63
**How disappointed were you? [n (%)]**	150 (45)	79 (24)	37 (11)	35 (10)	28 (8)	6 (2)	
1 cancellation **[n (%)]**	72 (41)	46 (26)	20 (11)	19 (11)	15 (9)	4 (2)	
>1 cancellation **[n (%)]**	78 (49)	33 (21)	17(11)	16(10)	13(8)	2(1)	0.73
**How angry were you? [n (%)]**	107 (32)	41 (12)	45 (13)	47 (14)	88 (26)	10 (3)	
1 cancellation **[n (%)]**	51 (29)	24 (13)	25 (14)	24 (13)	50 (28)	4 (2)	
>1 cancellation **[n (%)]**	56 (35)	17 (11)	20 (13)	23 (14)	38 (24)	6 (4)	0.69
**How frustrated were you? [n (%)]**	139 (41)	58 (17)	42 (12)	52 (15)	36 (11)	12 (4)	
1 cancellation **[n (%)]**	70 (39)	36 (20)	20 (11)	30 (17)	19 (11)	5 (3)	
>1 cancellation **[n (%)]**	69 (43)	22 (14)	22 (14)	22 (14)	17 (11)	7 (4)	0.57
**How stressed were you? [n (%)]**	129 (38)	49 (14)	41 (12)	42 (12)	71 (21)	7 (2)	
1 cancellation **[n (%)]**	67 (36)	28 (15)	22 (12)	26 (14)	41 (22)	2 (1)	
>1 cancellation **[n (%)]**	62 (41)	21 (14)	19 (12)	16 (10)	30 (20)	5 (3)	0.61

For peer review only

Table 3: Sample of free text comments from responders

"I was aware of winter pressures and was expecting op to be cancelled. Felt it was right someone with greater need took priority."
"As I am on my own, with no family nearby, it had no real impact. Just a few phone calls to cancel lifts in and back. New appointment for 4th December, so not long to wait. Quite understood situation with the start of the "winter strain", so no point in getting stressed. No complaints at all."
"Struggling to swallow, risk of choking when eating."
"The surgeon was ready to do my operation on the correct day but was told at the last minute there was no bed for me in the ICU/high dependency ward so he couldn't do it. I was very disappointed, but it was not the surgeon's fault."
"I totally understood why my operation was cancelled. My operation needed to be done but my life was not in danger. It was just keeping my work informed so cover could be found whilst I was off work"
"It was cancelled the day before. This meant I had to re-arrange work, so did my partner and the childcare for 2 days. All at late notice. We all work full time. I was also due to fly to America for work and this now became an issue."
"I was disappointed on the original date being cancelled and postponed by 9/10 weeks. It was not a very serious lift threatening situation for me and there were other serious cancer operations that required operations so I fully understood the reasons etc."

For peer review only - http://bmjopen.bmj.com/site/about/guidelines.xhtml

“Obviously at the time I felt sad because you not only gear yourself up to have the operation, but I could not justify taking up a hospital bed when there were more needy cases than mine requiring treatment. I do feel more money and less stress for NHS workers is required and the government needs to take heed!”

“I was waiting 6 months for this operation. This meant our lives were "put on hold" for this time. We could not book holidays, arrange anything that involved being away from home as we were expecting op dates; no information on delay times was given. This is very stressful”

“I was surprised to be given pre-med drugs before being told the operation was cancelled. I left the hospital feeling "spaced out"!”

“Each time I came for pre op washout the day before to be told when called in that there is no operation tomorrow. I have a young family so I have to prepare them emotionally.”

“I am usually active and my hobbies and enjoyment usually is by way of playing sport. Not being able to undertake these activities had the obvious physical affects and resulted in me being in a low mood, even feeling depressed at times. In turn, I found myself comfort eating and drinking more alcohol. In conclusion, the physical and mental effects were profound.”

“Operation performed 5 weeks later, did not affect outcome.”

“Cancelled holiday. Developed sepsis after being discharged.”

“Very disappointed.”

“Such late notice when each operation was cancelled (day before). Very mentally draining preparing for our op then it getting cancelled.”

For peer review only

"I was given a date. When I reached hospital there was no bed. Cancellation happened three times"

"My operation was cancelled due to a trauma case on that day. Everyone at the hospital was so kind and op was arranged for the next week. Thank you."

"My biggest problem has been the effect the original cancellation had on my elderly mother (aged 97) who was booked into respite care for six weeks @ £500 per week. She is still there some six months later. Her chances of coming home have diminished to almost none."

"It was rubbish at the time but completely understandable. Government should take the hit not the NHS. You guys are great and do all you can!"

"I was very distressed, I cried frequently and was very anxious. I felt let down and became depressed. I have not felt like doing anything and have rarely left the house. My irritable bowel syndrome got worse and my mental health has not been good and I have to take medication. My relationships have become strained and I feel I can't trust people."

"It made me feel that I didn't really matter! Although my condition is not life threatening, it is life debilitating."

For peer review only

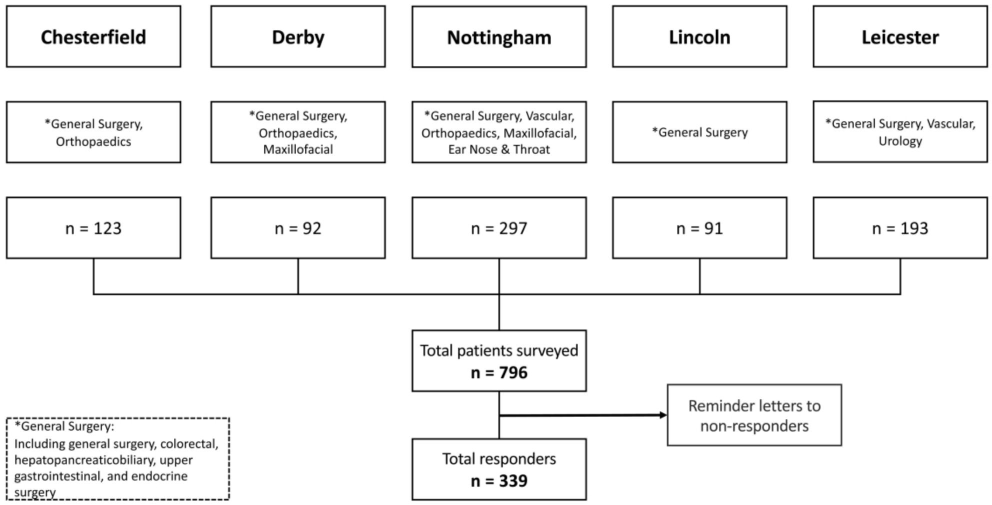

Figure 1: Study flow diagram.

282x144mm (300 x 300 DPI)

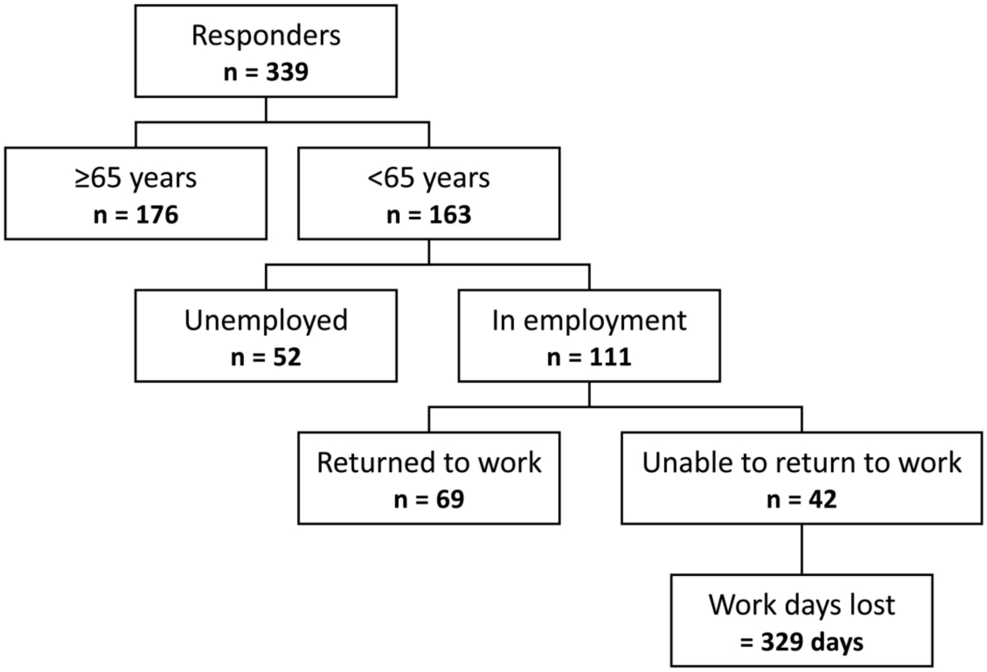

Figure 2: Number of work days lost.

188x129mm (300 x 300 DPI)

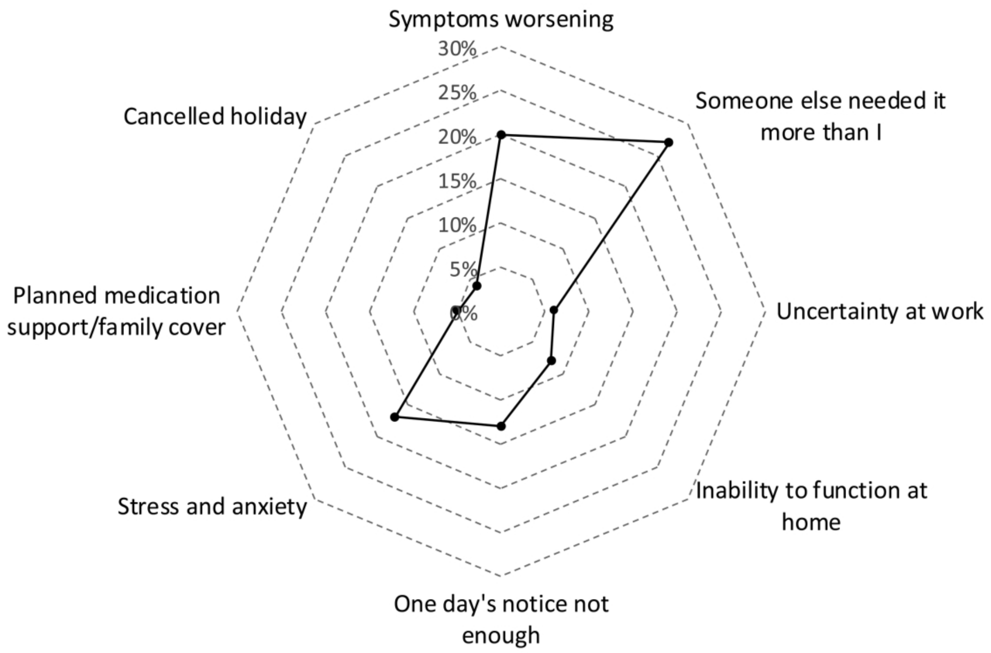

Figure 3: Radar diagram of thematic analysis (figures represent percentage of responders).

231x150mm (300 x 300 DPI)

emsan.co.uk/wespi wespistudy@gmail.com

Winter Elective Surgery Cancellation and Psychological Impact survey

WES-Pi

Please tell us about you:

Q1) What is your employment status?
*Please select **one** option only.*

- Employed - full time ☐
- Employed - part time ☐
- Self-employed - full time ☐
- Self-employed - part time ☐
- In education - full time ☐
- In education - part time ☐
- Unemployed ☐
- Retired ☐

Q2) What is your age group?
*Please select **one** option only.*

- 16-24 years ☐
- 25-34 years ☐
- 35-44 years ☐
- 45-54 years ☐
- 55-64 years ☐
- 65-74 years ☐
- 75-84 years ☐
- Over 85 years ☐
- Prefer not to say ☐

Q3) On what date was your operation meant to be?
Please provide an estimate if you are unsure of the exact date.

Date: d d - m m - y y y y

Q4) On what date were you told your operation was cancelled or postponed?
Please provide an estimate if you are unsure of the exact date.

Date: d d - m m - y y y y

Q5) If your operation was cancelled or postponed previously, how many times did this occur in total?
Please provide an estimate if you are unsure of the exact number of times.

[ ] times

For peer review only - http://bmjopen.bmj.com/site/about/guidelines.xhtml

Q6) What operation were you meant to be having?

Please describe in your own words, e.g. hernia operation, hip replacement, etc.

Please tell us about the impact of your operation being cancelled or postponed on your work and finances:

Q7) Due to your operation being cancelled or postponed, were you able to **go to work** instead?

Please provide an estimate of the days you think you needed to take off work.

Yes ☐
No* ☐
Not applicable ☐

*If not, how many **days off** did you take?

Q8) Do you think you will need **extra days off work** because your operation was cancelled or postponed?

Please provide an estimate of the day/s you think you will need off work, due to your untreated condition preventing you from working, or the additional time off you will have to take when you receive another date for your operation.

Yes* ☐
No ☐
Not applicable ☐

*If yes, how many **day/s off**?

Q9) Did your friends or family members have to take **extra days off work** because your operation was cancelled or postponed?

Please provide an estimate of the day/s your friends or family members have taken so far, to either look after you when unwell (because you did not have your operation), or planned to take off to look after you following your operation

Yes* ☐
No ☐
Not applicable ☐

*If yes, how many **day/s off**?

For peer review only - http://bmjopen.bmj.com/site/about/guidelines.xhtml

Q10) If you are a parent or carer, have <u>you</u> needed **extra childcare** or **carer support**, because your operation was cancelled or postponed?

Please provide an estimate of the total time you have needed so far. e.g. having to book extra support when you are unwell (because you did not have your operation), or needing to rebook extra support for when you receive another operation date.

Yes* ☐
No ☐
Not applicable ☐

*If yes, how many **hours*** or **days***?
****please circle as appropriate***

Q11) Have <u>you</u> had **extra costs in travel and parking** because your operation was cancelled or postponed?

Please provide an estimate if you are unsure of the exact costs so far. e.g. additional journeys to the hospital/doctors because you have been unwell (because you did not have your operation), or because you have needed extra tests before receiving another operation date.

Yes* ☐
No ☐
Not applicable ☐

If yes, how much **extra cost**?

Please tell us about the impact of your operation being cancelled or postponed on your <u>mental health and well-being</u>:

Q12) Are there any key **social activities/hobbies** <u>you</u> have had to miss because your operation was cancelled or postponed?

These may include family days out, attendance at weddings, visiting friends, or your ability to physically participate in your hobbies or interests outside of work.

Yes* ☐
No ☐
Not applicable ☐

*If yes, is there anything specific you would like to mention?

For peer review only - http://bmjopen.bmj.com/site/about/guidelines.xhtml

For each question please select **one** option that best describes **your feelings, <u>at the time</u>** your operation was cancelled or postponed:

	Not at all	Slightly	Moderately	Very	Extremely	Not applicable
Q13) How **sad** were you?	☐	☐	☐	☐	☐	☐
Q14) How **disappointed** were you?	☐	☐	☐	☐	☐	☐
Q15) How **angry or annoyed** were you?	☐	☐	☐	☐	☐	☐
Q16) How **frustrated** were you?	☐	☐	☐	☐	☐	☐
Q17) How **stressed** were you?	☐	☐	☐	☐	☐	☐

For each question please select **one** option that best describes **your concerns, <u>at the time</u>** your operation was cancelled or postponed:

	Not at all	Slightly	Moderately	Very	Extremely	Not applicable
Q18) How concerned were you about your **symptoms continuing**?	☐	☐	☐	☐	☐	☐
Q19) How concerned were you about your **health deteriorating**?	☐	☐	☐	☐	☐	☐

Q20) Is there anything else you would like to mention about how your operation being cancelled or postponed has affected you?
e.g. how it made you feel, how it impacted on your life, how it affected you physically, whether you were given enough information, etc.

Thank you for taking the time to complete this survey

Please return it in the pre-paid envelope provided within **<u>two weeks</u>** of receipt.

For peer review only - http://bmjopen.bmj.com/site/about/guidelines.xhtml

National Institute for Health Research

WES-Pi

1st March 2018

Dear Patient,

As you may be aware, due to the increased pressures faced by the NHS during the winter period, some patients like yourself have unfortunately had their operations either cancelled or delayed.

To understand the true impact this can have on our patients' lives, we are asking all patients who have experienced a cancellation or delay to complete the attached short survey (Winter Elective Surgery Cancellation and Psychological Impact survey).

You do not have to participate in this survey, but your experiences may help us to improve our services for future patients. There are no right or wrong answers to any of the questions asked, it is your opinions that we are really interested in. In particular, we would like to hear how your experience of a cancellation has affected you emotionally, physically, and financially.

You do not have to give any personal details, and the results of the survey will be completely anonymous when published. To participate, please fill in the attached survey and return it to us in the pre-paid envelope provided. If you have any further questions about this survey you can contact us at: wespistudy@gmail.com

Thank you for your time.

Yours sincerely,

The East Midlands Surgical Academic Network (EMSAN)

EMSAN is a group of trainee doctors who work for the NHS. We believe in making research opportunities available to everyone, to aid learning and improve medical services.

Visit us at: emsan.co.uk/wespi
Contact us at: wespistudy@gmail.com

For peer review only

Patient Public Involvement in study survey design

Question No.	Question	PPI Lay Assessors – random comments	Study lead response
Q5	In your own words, what operation were you meant to be having?	Wording change: "Had your operation been previously cancelled or postponed? If so, how many times?"	Amended
Q8	How many **extra** days off work do you now think **you** will miss because of the health condition that you were due to have the operation for?	This is a rhetorical question I doubt even Mr Adiamah could answer correctly.	This has now been amended to reflect the time taken for the surgery and immediately afterwards, and potential time taken off due to ongoing illness related to the cancellation
Q10	How much extra cost has **childcare** cost you because of your operation being cancelled or postponed?	Question on childcare costs. May not be relevant to all participants, and potentially quite intrusive. Also, costs can be very variable.<br><br>Suggestion of using 'hours of childcare' instead.	Question amended to ask about 'hours of childcare<br><br>AND carer support' if relevant.<br><br>Descriptor added.
Q13-Q18	Please answer the following questions in relation to your operation being cancelled or postponed.<br><br>*For each question please select ONE option that*	Surely 'today' might not be a good measure of feelings about the change in plan	Amended to ask about feelings at the time of the postponement instead of 'today'.

For peer review only

	best describes your feelings ***today.***<br><br>How **disappointed (Q13); angry (Q14); frustrated (Q15); stressed (Q16)** are you about your operation?<br><br>How concerned are you about<br><br>your **symptoms continuing**? (Q17); **health deteriorating**? (Q18)		
Overall	Additional box for other comments (this was suggested by two of the PPI Lay Assessors)		Great suggestion, thanks – we have added this.

For peer review only

STROBE Statement—checklist of items that should be included in reports of observational studies

	Item No	Recommendation	Page number
**Title and abstract**	1	(*a*) Indicate the study's design with a commonly used term in the title or the abstract	1
		(*b*) Provide in the abstract an informative and balanced summary of what was done and what was found	3
**Introduction**			
Background/rationale	2	Explain the scientific background and rationale for the investigation being reported	6-7
Objectives	3	State specific objectives, including any prespecified hypotheses	7
**Methods**			
Study design	4	Present key elements of study design early in the paper	8-12
Setting	5	Describe the setting, locations, and relevant dates, including periods of recruitment, exposure, follow-up, and data collection	8
Participants	6	(*a*) *Cohort study*—Give the eligibility criteria, and the sources and methods of selection of participants. Describe methods of follow-up<br>*Case-control study*—Give the eligibility criteria, and the sources and methods of case ascertainment and control selection. Give the rationale for the choice of cases and controls<br>*Cross-sectional study*—Give the eligibility criteria, and the sources and methods of selection of participants	8
		(*b*) *Cohort study*—For matched studies, give matching criteria and number of exposed and unexposed<br>*Case-control study*—For matched studies, give matching criteria and the number of controls per case	
Variables	7	Clearly define all outcomes, exposures, predictors, potential confounders, and effect modifiers. Give diagnostic criteria, if applicable	8-12
Data sources/ measurement	8*	For each variable of interest, give sources of data and details of methods of assessment (measurement). Describe comparability of assessment methods if there is more than one group	*8-12*
Bias	9	Describe any efforts to address potential sources of bias	9-12
Study size	10	Explain how the study size was arrived at	8
Quantitative variables	11	Explain how quantitative variables were handled in the analyses. If applicable, describe which groupings were chosen and why	12
Statistical methods	12	(*a*) Describe all statistical methods, including those used to control for confounding	12
		(*b*) Describe any methods used to examine subgroups and interactions	12
		(*c*) Explain how missing data were addressed	12
		(*d*) *Cohort study*—If applicable, explain how loss to follow-up was addressed<br>*Case-control study*—If applicable, explain how matching of cases and controls was addressed<br>*Cross-sectional study*—If applicable, describe analytical methods taking account of sampling strategy	8-12
		(*e*) Describe any sensitivity analyses	NA

For peer review only

Continued on next page

Results			Page number
Participants	13*	(a) Report numbers of individuals at each stage of study—eg numbers potentially eligible, examined for eligibility, confirmed eligible, included in the study, completing follow-up, and analysed	13
		(b) Give reasons for non-participation at each stage	13
		(c) Consider use of a flow diagram	Fig1
Descriptive data	14*	(a) Give characteristics of study participants (eg demographic, clinical, social) and information on exposures and potential confounders	13
		(b) Indicate number of participants with missing data for each variable of interest	13
		(c) *Cohort study*—Summarise follow-up time (eg, average and total amount)	
Outcome data	15*	*Cohort study*—Report numbers of outcome events or summary measures over time	
		Case-control study—Report numbers in each exposure category, or summary measures of exposure	*13*
		Cross-sectional study—Report numbers of outcome events or summary measures	
Main results	16	(*a*) Give unadjusted estimates and, if applicable, confounder-adjusted estimates and their precision (eg, 95% confidence interval). Make clear which confounders were adjusted for and why they were included	13-15
		(*b*) Report category boundaries when continuous variables were categorized	
		(*c*) If relevant, consider translating estimates of relative risk into absolute risk for a meaningful time period	
Other analyses	17	Report other analyses done—eg analyses of subgroups and interactions, and sensitivity analyses	13-15
**Discussion**			
Key results	18	Summarise key results with reference to study objectives	16-19
Limitations	19	Discuss limitations of the study, taking into account sources of potential bias or imprecision. Discuss both direction and magnitude of any potential bias	18
Interpretation	20	Give a cautious overall interpretation of results considering objectives, limitations, multiplicity of analyses, results from similar studies, and other relevant evidence	16-19
Generalisability	21	Discuss the generalisability (external validity) of the study results	16-19
**Other information**			
Funding	22	Give the source of funding and the role of the funders for the present study and, if applicable, for the original study on which the present article is based	N/A

*Give information separately for cases and controls in case-control studies and, if applicable, for exposed and unexposed groups in cohort and cross-sectional studies.

Note: An Explanation and Elaboration article discusses each checklist item and gives methodological background and published examples of transparent reporting. The STROBE checklist is best used in conjunction with this article (freely available on the Web sites of PLoS Medicine at http://www.plosmedicine.org/, Annals of Internal Medicine at http://www.annals.org/, and Epidemiology at http://www.epidem.com/). Information on the STROBE Initiative is available at www.strobe-statement.org.

For peer review only

BMJ Open

Winter cancellations of elective surgical procedures in the United Kingdom: A questionnaire survey of patients on the economic and psychological impact

Journal:	*BMJ Open*
Manuscript ID	bmjopen-2018-028753.R2
Article Type:	Research
Date Submitted by the Author:	16-Aug-2019
Complete List of Authors:	Herrod, Philip; Royal Derby Hospital, Surgery<br>Adiamah, Alfred; University of Nottingham<br>Boyd-Carson, Hannah; Royal Derby Hospital<br>Daliya, Prita; University of Nottingham<br>El-Sharkawy, Ahmed; Royal Derby Hospital<br>Sarmah, Panchali; University Hospitals of Leicester NHS Trust<br>Hossain, Tanvir; Nottingham University Hospitals NHS Trust<br>Couch, Jennifer; Nottingham University Hospitals NHS Trust, Department of General Surgery<br>Sian, Tanvir; Royal Derby Hospital<br>Wragg, Andrew; University of Nottingham, Nottingham Digestive Diseases Centre<br>Andrew, David; Lincoln County Hospital<br>Parsons, Simon; Nottingham University Hospitals NHS Trust, Department of General Surgery<br>Lobo, Dileep; University of Nottingham, Nottingham Digestive Diseases Centre
**Primary Subject Heading**:	Surgery
Secondary Subject Heading:	Qualitative research, Patient-centred medicine, Medical management
Keywords:	SURGERY, Adult surgery < SURGERY, QUALITATIVE RESEARCH, Organisation of health services < HEALTH SERVICES ADMINISTRATION & MANAGEMENT

SCHOLARONE™
Manuscripts

Winter cancellations of elective surgical procedures in the United Kingdom: A questionnaire survey of patients on the economic and psychological impact

Philip J J Herrod*[1,2], Alfred Adiamah*[1,2], Hannah Boyd-Carson[1,2], Prita Daliya[1,2], Ahmed M El-Sharkawy[1,2], Panchali B Sarmah[1,3], Tanvir Hossain[1,2], Jennifer Couch[1,2], Tanvir S Sian[1,2], Andrew Wragg[4], David R Andrew[5], Simon L Parsons[1,2], Dileep N Lobo[1,2,6], WES-Pi Study Group on behalf of the East Midlands Surgical Academic Network (EMSAN)†

*Joint first authors

[1]East Midlands Surgical Academic Network, Queen's Medical Centre, NG7 2UH, UK
[2]Gastrointestinal Surgery, Nottingham Digestive Diseases Centre and National Institute for Health Research (NIHR) Nottingham Biomedical Research Centre, Nottingham University Hospitals and University of Nottingham, Queen's Medical Centre, Nottingham NG7 2UH, UK
[3]Leicester Cancer Research Centre, University of Leicester, LE2 7LX, UK
[4]Patient & Public Involvement Facilitator, Gastrointestinal and Liver Disorders Theme, National Institute for Health Research (NIHR) Nottingham Biomedical Research Centre, Nottingham University Hospitals NHS Trust and the University of Nottingham, Queen's Medical Centre, Nottingham NG7 2UH, UK
[5]Lincoln County Hospital, Greetwell Road, Lincoln LN2 5QY, UK
[6]MRC/ARUK Centre for Musculoskeletal Ageing Research, School of Life Sciences, University of Nottingham, Queen's Medical Centre, Nottingham NG7 2UK, UK

† **WES-Pi Study Group:**

Jennifer Couch, Prita Daliya, Tanvir Hossain, Bethan Johnson, Amanda Koh, Anisa Kushairi, Dileep N Lobo, Simon L Parsons, Andrew Wragg (Nottingham University Hospitals NHS Trust); Alfred Adiamah, David R Andrew, Chris Lewis-Lloyd, Farah Roslan, Amari Thompson, Helen Wan (United Lincolnshire Hospitals NHS Trust); Ahmed M El-Sharkawy, Sita Kotecha, Kevin Sargen (Chesterfield Royal Hospitals NHS Foundation Trust); Hannah Boyd-Carson, Phillip J J Herrod, Jonathan N Lund, Alastair Morton, Tanvir S Sian (Derby Teaching Hospitals NHS Foundation Trust); Edmund Charles, Panchali B Sarmah, Baljit Singh (University Hospitals of Leicester NHS Trust).

Address for correspondence:
Professor Dileep N Lobo
Gastrointestinal Surgery
Nottingham Digestive Diseases Centre
Nottingham University Hospitals NHS Trust and University of Nottingham
E Floor, West Block
Queen's Medical Centre
Nottingham NG7 2UH, UK
Tel: +44-115-8231149
Fax: +44-115-8231160
E-mail: Dileep.Lobo@nottingham.ac.uk

For peer review only

Category: Original research

Word count (excluding title page, abstract, figure legends, tables and references): 3340

Table and figures: 3 tables and 3 figures + 2 supplementary digital documents

Running head: Winter Elective Surgery Cancellation and Psychological Impact (WES-Pi) Survey

Key words: winter cancellations; elective operations; psychosocial impact; economic impact; National Health Service

Funding: This work was supported by the Medical Research Council [grant number MR/K00414X/1]; and Arthritis Research UK [grant number 19891]. AA was funded by a National Institute for Health Research (NIHR) Academic Clinical Fellowship.

The funders had no role in the design or conduct of the work, or in the decision to publish. This paper presents independent research funded by the MRC, ARUK and the NIHR. The views expressed are those of the authors and not necessarily those of the MRC, ARUK, NHS, the NIHR or the Department of Health.

Conflict of interest: None of the authors has a direct conflict of interest to declare. SLP is a company director for EIDO Healthcare Limited (unrelated to the submission). DNL has received an unrestricted research grants for unrelated work from BBraun in the last 3 years. He has also received speakers' honoraria from BBraun, Fresenius Kabi, Shire and Baxter Healthcare for unrelated work in the last 3 years.

This paper was to the Annual Meeting of the Society for Academic and Research Surgery in London, UK on 8 January 2019 and has published in abstract form in the *British Journal of Surgery* (Br J Surg 2019;106(S3):31).

"The Corresponding Author has the right to grant on behalf of all authors and does grant on behalf of all authors, a worldwide licence to the Publishers and its licensees in perpetuity, in all forms, formats and media (whether known now or created in the future), to i) publish, reproduce, distribute, display and store the Contribution, ii) translate the Contribution into other languages, create adaptations, reprints, include within collections and create summaries, extracts and/or, abstracts of the Contribution, iii) create any other derivative work(s) based on the Contribution, iv) to exploit all subsidiary rights in the Contribution, v) the inclusion of electronic links from the Contribution to third party material where-ever it may be located; and, vi) licence any third party to do any or all of the above."

For peer review only

ABSTRACT

Objectives: To quantify the economic and psychological impact of the cancellation of operations due to winter pressures on patients, their families and the economy.

Design: This questionnaire study was designed with the help of patient groups. Data were collected on the economic and financial burden of cancellations. Emotions were also quantified on a 5-point Likert scale.

Setting: Five NHS Hospital Trusts in the East Midlands region of England.

Participants: We identified 796 participants who had their elective operations cancelled between 1-11-2017 and 31-3-2018 and received responses from 339 (43%).

Interventions: Participants were posted a modified version of a validated quality of life questionnaire with a pre-paid return envelope.

Main Outcome Measures: The primary outcome measures were the financial and psychological impact of the cancellation of elective surgery on patients and their families.

Results: Of the 349 respondents, 163 (48%) were aged <65 years, with 111 (68%) being in employment. Sixty-six (19%) had their operations cancelled on the day. Only 69 (62%) of working adults were able to return to work during the time scheduled for their operation, with a mean (standard deviation, SD) loss of 5 working days (SD 10). Additional working days were lost subsequently by 60 (54%) participants [mean 7 days (SD 10)]. Family members of 111 (33%) patients required additional time off work [mean 5 days (SD 7)]. Over 30% of participants reported extreme levels of sadness, disappointment, anger, frustration and stress. At least moderate concern about continued symptoms was reported by 234 (70%)

For peer review only

participants, and 193 (59%) reported at least moderate concern about their condition deteriorating.

Conclusions: The cancellation of elective surgery during the winter had an adverse impact on patients and the economy, including days of work lost and health-related anxiety. We recommend better planning, and provision of more notice and better support to patients.

For peer review only

Strengths

- This study presents aggregated evidence to support previous anecdotes of the importance of cancellation of elective operations to patients' well-being. It was devised with active consultation with patient groups. The questions asked were those that the patient groups thought relevant to the problem.
- It highlights the importance of early notification of cancellations in times such as winter pressures, where cancellations are unavoidable to sustain the delivery of emergency care.

Limitations

- This study was retrospective by design and was potentially limited by recall bias
- We achieved a less than 50% responder rate, in spite of two rounds of questionnaires being sent, and as such is subject to non-responder bias.
- The study covered a single but large region of the United Kingdom, it is difficult to ascertain if the population dynamics and level of social deprivation make this population similar to those in all parts of the UK, and as such affects the generalisability of our results.

For peer review only

INTRODUCTION

The winter of 2017-2018 saw an unsustainable increase in patient demand on acute hospital care in England, due both to an anticipated seasonal rise in admissions and an unexpectedly severe epidemic of winter flu. In response to what was dubbed the "Winter National Health Service (NHS) Crisis" by the media, NHS England took the unprecedented step of instructing all NHS hospital trusts to cancel planned routine non-cancer surgery throughout December 2017 and January 2018, in order to free resources for emergency admissions.[1]

Although official figures have not yet been released, media estimates put the number of patients affected in England at around 50,000.[2] This huge volume of cancellations will have ongoing ramifications for the 2018 calendar year.[3 4] The rescheduling of a large backlog of cancelled operations by hospital trusts, will undoubtedly put added pressure on hospital waiting lists.[3 4]

Despite the effect of a cancellation on the ongoing care provided by hospitals being subject to many reporting structures, the psychological and economic impact of a cancellation on patients themselves has received remarkably little attention. The requirement of a period of recuperation, means most patients in their preparation for elective surgery make arrangements in their social lives to facilitate this. Adults in employment may book time off work and postpone important social events such as holidays. Carers may similarly organise additional support or childcare arrangements. Family members may also do the same in order to help care for recovering patients. If a planned operation is postponed at short notice, it is possible to envisage these arrangements resulting in personal and economic loss.

For peer review only

Any rescheduled operative date may also result in patients and carers incurring the same costs again with no recourse to reimbursement. This may lead to a considerable economic burden on the patient and their family[5] and cause additional loss of working days for the economy. Postponement or cancellation of elective surgery may also have a profound psychological impact on patients and their families, who may have mentally prepared themselves for the event. There may also be significant health-related anxiety associated with a longer wait for surgery, as patients may fear their condition deteriorating further.[6] We aimed, in this **W**inter **E**lective **S**urgery Cancellation and **P**sychological **i**mpact (WES-Pi) survey, to quantify the financial and psychological impact on patients following the postponement or cancellation of elective surgery in England, due to winter pressures.

For peer review only

METHODS

This multicentre study was conducted across five NHS acute hospital trusts (Nottingham University Hospitals NHS Trust, University Hospitals of Leicester NHS Trust, Derby Teaching Hospitals NHS Foundation Trust, United Lincolnshire Hospitals NHS Trust and Chesterfield Royal Hospitals NHS Foundation Trust)in the East Midlands region of England during the winter of 2017-18 in accordance with the STROBE statement.[7] Adult patients aged 16 years or over, scheduled for elective surgery between 1 November 2017 and 31 March 2018 were identified from local hospital waiting list records. As elective paediatric is centralised and the majority of NHS Trusts contributing to this study do not provide a service, our focus was on adult patients only. Participants were chosen from the surgical subspecialties of general surgery, colorectal, hepatopancreaticobiliary, upper gastrointestinal, endocrine, vascular, urology, orthopaedics, maxillofacial, and ear nose and throat. All patients who had their elective surgery postponed or cancelled for non-patient related reasons were included (**Figure 1**). Patients who had surgery postponed or cancelled for patient-related reasons (e.g. unrelated illness, medically unfit, or surgery declined), or those that died before dispatch of the questionnaires were not invited to participate.

For peer review only

Outcomes

Our primary outcome measures were to quantify the financial and psychological impact of cancellation of elective surgery on participants and their families. The secondary outcome measure was to evaluate the impact of the length of notice of cancellation on the primary outcomes.

Survey design

In the absence of an existing validated questionnaire to explore financial or emotional consequences to a patient, specifically related to the cancellation of elective surgery, we modified a previously used survey from a paediatric study.[8] Survey design involved multiple iterations and amendments through patient focus groups and significant involvement and input from the Patient Advisory Group at Nottingham University Hospitals NHS trust, and the Patient Advisory and Liaison Service (PALS) team at Derby Teaching Hospitals NHS Trust. In addition to gathering data on the economic and financial burden of postponements and cancellations on participants and their relatives, the questionnaire also quantified emotional feelings of participants on a 5-point Likert scale[9] (**Appendix 1**).

Patient and public involvement

Patient and Public Involvement (PPI) was an integral element throughout this study given its direct relevance to patient care. Embedding PPI into to the study from the outset meant we could more accurately explore the patient experience of cancellations informed by what patients felt from prior experience were important in their care. There is a current drive for PPI in all medical research, for experiential reasons (Patients or carers have experience of a condition, treatment or in this instance experience of a cancelled appointment for elective operation); ethical and moral reasons (Patients and the public are directly affected by the research process and findings; they have the right to contribute to it; and of course are the tax payers); and pragmatic reasons (PPI is a requirement for funding bodies; it translates

For peer review only

research into knowledge). In this instance, we felt strongly that for a study where the purpose was solely about patient experience, it had to be informed fully by patients.

We chose to recruit PPI Lay Assessors to review the invitation letter and subsequent survey to be sent to study participants. We wanted to prove that the intent to use a survey to explore the impact of cancelled elective operations on the psychosocial wellbeing of our patients, as a means leading to service improvement, if suitably structured would achieve its outcomes. PPI provided assurance that the devised series of questions contained in the survey was suitable to cover the aspects of financial, psychological and physical impact of these cancellations including symptomatic (suggestive) deterioration without adversely impacting on patients or being too onerous to complete.

They directed the changes through several iterations and approved of the final survey and invitation letter, ensuring the overall suitability and acceptability of the survey.

How was the development of the research question and outcome measures informed by patients' priorities, experience, and preferences?

In consultation with PPI members, the research tool was shaped to more accurately assess the financial impact of cancellations, not only for patients but also for their family members involved in their care. In exploring patients' emotions, their responses allowed us to make the questions more specific to feelings at the time of cancellations (**Appendix 1**)

How did you involve patients in the design of this study?

For peer review only

From the PPI perspectives, the measures were informed via six (6) recruited PPI members from the Gastrointestinal & Liver Patient Advisory Group, charged with reviewing these study public-facing documents (invitation letter and survey to be issued to the study participants). Each lay PPI Member assessed and reported back their respective thoughts on the document(s) content, layout and its message. Examples of comments (on the survey) made, and responses made by study leads are presented in **Appendix 2**

Were patients involved in the recruitment to and conduct of the study?

No. Recruitment was informed by the cancellation list at each participating trust.

How will the results be disseminated to study participants?

The write up of the manuscript included the lead person from the Gastrointestinal and Liver PPI group, allowing the patient viewpoints to be more accurately reflected in our results. It also ensures dissemination to the initial group who were involved in the study design. More importantly there is an opportunity for this study to aid advocacy in the conduct of patient cancellations in a manner that results in the least adverse outcomes for patients.

For randomised controlled trials, was the burden of the intervention assessed by patients themselves?

Not applicable.

Ethics and Data collection

For peer review only

For peer review only - http://bmjopen.bmj.com/site/about/guidelines.xhtml

The study was registered and approved locally at each participating hospital trust, under the supervision of a named consultant surgeon as a service improvement project. The project registration/approval numbers for each site were: Nottingham University Hospitals NHS Trust 18-050c, University Hospitals of Leicester NHS Trust 9339E, Derby Teaching Hospitals NHS Foundation Trust SB-Gen-2017/18 -1025, United Lincolnshire Hospitals NHS Trust 1301 and Chesterfield Royal Hospitals NHS Foundation Trust WES-Pi 001. Identified participants were posted a copy of the study questionnaire (**Appendix 1**), a pre-paid return envelope, and an invitation letter (**Appendix 2**), incorporating local trust branding. Invitations were first posted to participants in April 2018, with a further invitation to non-responders in June 2018. Each study site was managed by local teams responsible for local study registration and approvals, identification of participants, dissemination of postal invitations, and data collection. Returned surveys were pseudoanonymised, with collated data subsequently anonymised and encrypted before central data analysis. No identifiable participant data were transferred between sites.

Data synthesis and analysis

Quantitative and qualitative data analyses of responses were undertaken. Quantitative data analysis was performed using STATA® v15.0 (StataCorp, College Station, TX, USA). Linear regression was performed to assess for correlation and a chi-square test to assess for differences between categorical data. All descriptive data are represented as n (%), mean (standard deviation, SD) or median (inter quartile range, IQR) as appropriate.

Qualitative data was evaluated using NVivo v11 (QSR International Inc., Burlington, MA, USA). For the purposes of thematic analysis, all the free text responses were transcribed and

For peer review only

data validated by two study authors independently. These were then coded and analysed using the NVivo software into 8 distinct themes that ranged from personal emotional response such as stress and anxiety; financial impact including cancelled holidays and time of work; and disease related concerns such as worsening of symptoms. The number of responses within each theme were then quantified and exported into excel and presented visually as a radar diagram.

For peer review only

For peer review only - http://bmjopen.bmj.com/site/about/guidelines.xhtml

RESULTS

Return rate and cancellation

Questionnaires were posted to 796 participants and returned by 339 (43%) after two distribution rounds. The response rate varied between trusts from 27% to 52%. Data on participant characteristics and surgical specialties involved are displayed in **Table 1**.

Of the participants returning questionnaires, 66 (19%) had their operations cancelled on the day of surgery, 44% (n=149) within 24 hours of the proposed surgery, and 37% (n=125) cancelled more than 48 hours from the proposed date of the operation. Median notification of cancellation was 2 days (IQR 1-6). A number of participants had been cancelled previously prior to the winter cancellation. The responders reported their operation as having been cancelled a median of 1 time (IQR 1-2) previously. In 15% (45/288) of the responders, operations had been cancelled three times or more (**Table 1**).

Economic burden

One hundred and sixty-three (48%) of responding participants were aged less than 65 years and of these 111 (68%) were in employment. Only 69 (62%) of the working adults were able to return to work during the time they had originally scheduled off for their operation, with a median loss of 1 day (IQR 0-5) of work. The total number of work days lost in the cohort was 329 (**Figure 2**).

Additional working days in subsequent months were lost by 60 (54%) of working adults due to the cancellation [median 3 days (IQR 0-12)], totalling 456 days. Family members of 111

For peer review only

(33%) participants required additional time off work median 3 days (IQR 1-5), totalling 581 days. Thus, the total number of missed days of work (from participants and family members, both from the initial cancellation and subsequent rescheduling of the operation) was 1366 days. There was no direct correlation between days of work lost and the notification given for cancellation (r^2=0.0004, p=0.87).

Child care costs and travel costs

Of the responders with children, 23 were forced to organise extra childcare. Of these the median (IQR) additional childcare days required was 4 days (0-22.5). One hundred and twenty-three participants (48%) incurred additional travel costs following cancellation of their operation. The median cost incurred for additional travel was £15 (IQR £6.5-£30).

Psychological burden

The majority of participants reported at least moderate levels of sadness, disappointment, anger, frustration and stress, with over 30% (n=102) of respondents reporting extreme levels of each of these emotions and over 50% of respondents reporting at least “very” (the second highest answer available) to each of these emotions. A full breakdown of emotions reported is displayed in **Table 2**. There was no significant difference in the reporting of these emotions between patients who had been cancelled only once or those who had suffered multiple cancellations. At least moderate concern about continued symptoms was reported by 234

For peer review only - http://bmjopen.bmj.com/site/about/guidelines.xhtml

(70%) patients whilst waiting for a rescheduled date whilst 193 (59%) reported at least moderate concern about their condition deteriorating.

There was no correlation between strength of any emotion and the amount of notice given to participants for the cancellation. This was true for all the emotive questions asked.

Thematic analysis from free text responses

The qualitative analysis of the participants' free text responses is presented in the thematic analysis as a radar diagram (**Figure 3**). Four particular themes were highlighted in this analysis. There was concern over symptoms worsening; stress and anxiety related to the cancellation; and those whose operations were cancelled on the day felt that this was unsatisfactory and caused them undue distress. However, the most interesting finding on the free text analysis was that 26% (70/268) of the responders understood the need for their operations to be cancelled to facilitate delivery of the emergency service. They used such descriptions as: "my operation was not life-threatening" and "others needed the care more" (**Table 3**).

For peer review only

DISCUSSION

The uncharacteristically cold weather, the high rates of emergency admissions, particularly in the elderly, and a lack of capacity in an already overworked and overstretched system gave rise to the perfect storm of cancellations during the winter of Nov 2017 to Feb 2018. Some even referred to it as another winter of discontent for the NHS.[10] The national strategy, instigated by NHS England was to cancel elective operations to release capacity in the system.[1] These elective cancellations were well publicised in the media, and meant that the majority of the population had some idea of the unusual stress the NHS was under.[10 11] However, despite public awareness, the impact on patients of these cancellations and, in particular last minute cancellations, was significant.

What this study found

This study is the first in England to evaluate the impact of the mass cancellation of elective surgery on patients, from both an economic and psychological view point. We have demonstrated that a large proportion of patients suffered a negative economic impact from both the additional work days lost and the additional non-refundable travel and childcare costs. We achieved this using a novel research tool, developed jointly with patient groups to establish usability, acceptability and internal validity.

Taken in the context of a nation-wide bed pressure crisis, which was widely publicised in the media, the fact that nearly 20% of patients had their operations cancelled on the day of surgery was surprising. Despite this we were unable to demonstrate any correlation between the period of notice given for cancellation of the planned operation, and the number of working days lost, nor any impact on the responses that patients gave to the

For peer review only

questions on their emotional state. Although the reasons behind this are unclear, our study may have been underpowered to demonstrate this and also due to the over representation of retired patients in our cohort.

Our relatively small sample from one region of the UK, comprising 5 hospitals, contained nearly 800 cancelled operations and in those responders led to the loss of 1366 work days to the economy from our modest 43% response rate. Given that this crisis stretched to the whole length of the UK and was estimated to have led to 50,000 cancellations nationwide, the projected loss of working days may exceed 85,000 days, even on the unlikely assumption that the non-responding patients lost no days from work. The questions on psychological impact, demonstrated that elective surgical cancellation has led to a large amount of negative emotion amongst patients, with the majority displaying at least a moderate amount of health-related anxiety.

Although this study was about winter cancellations of operations of patients from one region of England, the results are probably generalisable to the rest of the UK and also to other countries. Moreover, the results are not just applicable to winter cancellations, but could be applied to cancellations occurring at other times of the year as well.

What is available in the literature?

Whilst several studies have evaluated the causes of bed pressures and failures of hospital trusts to meet the emergency department 4-hour performance target[11 12] and others have evaluated the reason for elective cancellations across a range of health care settings,[13 14] very little information is available on the psychosocial impact of cancellations of elective operations on patients. Others have reported on measures to reduce same day cancellations

For peer review only - http://bmjopen.bmj.com/site/about/guidelines.xhtml

of elective operations, but have focused on other aspects, such as ensuring fitness for surgery, preventing the scheduling of the wrong operation, or preventing incomplete preoperative assessments rather than addressing cancellations due to a lack of beds.[15] Others have counted the cost of such last minute cancellations to the hospital itself, from specialised equipment that might need to be brought in, and noted that cancellations within 24 hours impacted significantly on costs to the trust.[16] In this study we found that 19% of the patients had their operations cancelled within 24 hours. Whilst this resulted in a financial impact on the patients, such late cancellations are also likely to increase costs for the hospital trust itself. From the point of view of optimal deployment of personnel and resources, better planning is needed for future crises. The Royal College of Surgeons of England has published guidance to help manage winter pressures and has acknowledged that it was better for patients to be told in advance of their cancellations in agreement with the findings of this study.[17] Additionally, they acknowledged the consequences of such cancellations inevitably would include prolonged suffering of the patients affected.[17] Better forecast and long-term planning of population health needs by policy makers are the only ways to avert these winter cancellations from becoming a recurring theme in the delivery of healthcare in the UK.

Limitations

Our study may be limited by its response rate, potential recall bias and non-responder bias. We took steps to maximise our response rate by sending pre-paid return envelopes with each questionnaire, and by dispatching a second round of questionnaires to non-responders. However, despite these efforts we were unable to improve our response rate above 50%. Unfortunately, as we did not receive any response at all from our non-responders, we do

For peer review only - http://bmjopen.bmj.com/site/about/guidelines.xhtml

know the reasons for the lack of response. We, therefore, cannot exclude that non-response bias has not led to an exaggeration of the proportion of patients report with negative economic or psychological impacts. However, we would contend, that even with the unlikely assumption that the non-responders did not experience any negative emotion or loss of work, the economic and psychological burden on this cohort was considerable. Another major limitation of our study was the lack of a pre-existing, validated survey tool. In order to mitigate against this, we based our survey design on a previously existing survey for another population, modified in conjunction with PPI involvement from two hospital trusts.

CONCLUSIONS

The WES-Pi survey has provided evidence to support the often-cited anecdotes concerning the impact of cancelling elective operations on patients' finances and psychological wellbeing. The national awareness of cancellations meant that the majority of responding patients understood the need for these cancellations. However, with 20% of respondents having their operations cancelled on the day of the procedure better planning is needed in the future to reduce cancellations and to provide more notice and better support to patients who have their operations cancelled. This may require a national change in policy by the Department of Health, in collaboration with specialist clinical commissioning groups and local trusts.

For peer review only - http://bmjopen.bmj.com/site/about/guidelines.xhtml

REFERENCES

1. Campbell D, Duncan P. NHS cancels surgery for tens of thousands to avoid winter crisis. *The Guardian* 21 December, 2017. Available at https://www.theguardian.com/society/2017/dec/21/nhs-cancels-surgery-tens-of-thousands-avoid-winter-crisis Accessed 10 November 2018.
2. Donnelly L, Bodkin H. NHS hospitals ordered to cancel all routine operations in January as flu spike and bed shortages lead to A&E crisis. *The Telegraph* 2 January, 2018. Available at https://www.telegraph.co.uk/news/2018/01/02/nhs-hospitals-ordered-cancel-routine-operations-january/ Accessed 10 November 2018.
3. Royal College of Surgeons of England. RCS: Last minute cancelled operations hits highest number since 1994. 10 May, 2018. Available at https://www.rcseng.ac.uk/news-and-events/media-centre/press-releases/nhs-performance-march-2018/ Accessed 12 August 2019.
4. Lintern S. Trust cancels operations until Easter to 'reset' health economy. *HSJ* 22 March, 2018. Available at: https://www.hsj.co.uk/nottingham-university-hospitals-nhs-trust/trust-cancels-operations-until-easter-to-reset-health-economy/7022000.article Accessed 12 August 2019.
5. Lankoandé M, Bonkoungou P, K Ki B, et al. Economic and psychological burden of scheduled surgery cancellation in a sub-Saharan country (Burkina Faso). Southern African Journal of Anaesthesia and Analgesia 2017;**23**(6):145-51.
6. Carr T, Teucher U, Mann J, et al. Waiting for surgery from the patient perspective. Psychol Res Behav Manag 2009;**2**:107-19.
7. von Elm E, Altman DG, Egger M, et al. Strengthening the Reporting of Observational Studies in Epidemiology (STROBE) statement: guidelines for reporting observational studies. BMJ 2007;**335**(7624):806-8.
8. Tait AR, Voepel-Lewis T, Munro HM, et al. Cancellation of pediatric outpatient surgery: economic and emotional implications for patients and their families. J Clin Anesth 1997;**9**(3):213-9.
9. Sullivan GM, Artino AR, Jr. Analyzing and interpreting data from likert-type scales. J Grad Med Educ 2013;**5**(4):541-2.
10. Campbell G. WINTER OF DISCONTENT? Plan to avoid hospital crisis at Christmas as pressure on A&E increases. *Oxford Mail* 17 November, 2017. Available at https://www.oxfordmail.co.uk/news/15666401.winter-of-discontent-plan-to-avoid-hospital-crisis-at-christmas-as-pressure-on-ae-increases/ Accessed 10 November 2018.
11. Keogh B, Culliford D, Guerrero-Luduena R, et al. Exploring emergency department 4-hour target performance and cancelled elective operations: a regression analysis of routinely collected and openly reported NHS trust data. BMJ Open 2018;**8**(5):e020296.
12. Morris AJ, McAvoy J, Dweik D, et al. Cancellation of elective cases in a recently opened, tertiary/quaternary-level hospital in the Middle East. Anesth Analg 2017;**125**(1):268-71.
13. Desta M, Manaye A, Tefera A, et al. Incidence and causes of cancellations of elective operation on the intended day of surgery at a tertiary referral academic medical center in Ethiopia. Patient Saf Surg 2018;**12**:25.

For peer review only

14. Kumar R, Gandhi R. Reasons for cancellation of operation on the day of intended surgery in a multidisciplinary 500 bedded hospital. J Anaesthesiol Clin Pharmacol 2012;**28**(1):66-9.
15. McKendrick DR, Cumming GP, Lee AJ. A 5-year observational study of cancellations in the operating room: Does the introduction of preoperative preparation have an impact? Saudi J Anaesth 2014;**8**(Suppl 1):S8-S14.
16. Sanjay P, Dodds A, Miller E, Arumugam PJ, Woodward A. Cancelled elective operations: an observational study from a district general hospital. J Health Organ Manag 2007;**21**(1):54-58.
17. Royal College of Surgeons of England. Guidance to help manage winter pressures. 28 November, 2018. Available at https://www.rcseng.ac.uk/news-and-events/news/archive/guidance-to-help-manage-winter-pressures/ Accessed 12 December 2018.

For peer review only

Statements

Competing interest statement

All authors have completed the Unified Competing Interest form at http://www.icmje.org/coi_disclosure.pdf (available on request from the corresponding author) and declare: no support from any organisation for the submitted work; no financial relationships with any organisations that might have an interest in the submitted work in the previous three years, no other relationships or activities that could appear to have influenced the submitted work." Please see http://resources.bmj.com/bmj/authors/editorial-policies/competing-interests

Exclusive licence

The Corresponding Author has the right to grant on behalf of all authors and does grant on behalf of all authors, an exclusive licence (or non exclusive for government employees) on a worldwide basis to the BMJ Publishing Group Ltd and its Licensees to permit this article (if accepted) to be published in BMJ editions and any other BMJPGL products and sublicences to exploit all subsidiary rights, as set out in our licence)."

Contributorship statement

Study design: PJJH, AA, HBC, PD, AME-S, PBS, TH, JC, TSS, DRA, AW, SLP, DNL

Development of questionnaire: AA, PJJH, PD, HBC, AW

Data collection: PJJH, AA, HBC, PD, AME-S, PBS, TH, JC, TSS, DRA, AW, SLP, DNL

Data-analysis: PJJH, AA, HBC

Data-interpretation: PJJH, AA, HBC, PD, DNL

Writing of Manuscript: PJJH, AA, PD, HBC, DNL

Creation of figures: AA, PD, DNL

Critical review of Manuscript: DRA, AW, SLP, DNL

Final approval: PJJH, AA, HBC, PD, AME-S, PBS, TH, JC, TSS, DRA, AW, SLP, DNL

PPI member's acknowledgements

We are very grateful to the following PPI members from the Gastrointestinal & Liver Patient Advisory Group, who worked on the questionnaire tool and whose contributions overall

For peer review only

shaped the study design. They are: Karen Andrews; David Harrison; David Matthews; John Shaw; Sally Taylor; and Stevie Vanhegan.

Guarantor Statement

The lead author (the manuscript's guarantor) affirms that the manuscript is an honest, accurate, and transparent account of the study being reported; that no important aspects of the study have been omitted; and that any discrepancies from the study as planned (and, if relevant, registered) have been explained.

Ethical approval

The study was registered and approved locally at each participating hospital trust, under the supervision of a named consultant surgeon as a service improvement project. The project registration/approval numbers for each site were: Nottingham University Hospitals NHS Trust 18-050c, University Hospitals of Leicester NHS Trust 9339E, Derby Teaching Hospitals NHS Foundation Trust SB-Gen-2017/18 -1025, United Lincolnshire Hospitals NHS Trust 1301 and Chesterfield Royal Hospitals NHS Foundation Trust WES-Pi 001.

Data sharing

Data will be available upon reasonable request from Mr. Philip J J Herrod (pherrod@nhs.net).

Patient involvement

The survey design involved multiple iterations and amendments through patient focus groups and significant involvement and input from the Patient Advisory Group at Nottingham University Hospitals NHS trust, and the Patient Advisory and Liaison Service (PALS) team at Derby Teaching Hospitals NHS Trust. One of the authors (AW) is a Patient & Public Involvement Facilitator.

For peer review only

LEGENDS FOR FIGURES

Figure 1: Study flow diagram.

Figure 2: Number of work days lost.

Figure 3: Radar diagram of thematic analysis (figures represent percentage of responders).

For peer review only

Table 1: Characteristics of the participants

Characteristic		Number (n)	%
**Age**	<65yrs	176	51.9
	≥65	163	48.1
**Employment status**	Employed	98	28.9
	Self Employed	23	6.8
	Retired	192	56.6
	Unemployed	26	7.7
**Previous cancellation history**	No cancellations	72	25.0
	1 cancellation	106	36.8
	2 cancellations	65	22.6
	≥3 cancellations	45	15.6
**Operations**	General* & Vascular	163	48.1
	Orthopaedic	135	39.8
	Others**	41	12.1
**Returns per hospital**	Nottingham	157	46.3
	Derby	42	12.4
	Chesterfield	42	12.4
	Lincoln	45	13.3
	Leicester	53	15.6

General*: Including general surgery, colorectal, hepatopancreaticobiliary, upper gastrointestinal and endocrine surgery.

Others**: Urology, ENT (ear, nose & throat surgery) and Maxillofacial surgery

For peer review only

Table 2: Responses to questions on psychological impact

	Extremely	Very	Moderately	Slightly	Not at all	Not Applicable	P
**How sad were you? [Total n (%)]**	107 (32)	56 (17)	45 (13)	70 (21)	40 (12)	21 (6)	
1 cancellation **[n (%)]**	54 (30)	27 (15)	28 (15)	41 (23)	21 (12)	10 (6)	
>1 cancellation **[n (%)]**	53 (34)	29 (18)	17 (11)	29 (18)	19 (12)	11 (7)	0.63
**How disappointed were you? [n (%)]**	150 (45)	79 (24)	37 (11)	35 (10)	28 (8)	6 (2)	
1 cancellation **[n (%)]**	72 (41)	46 (26)	20 (11)	19 (11)	15 (9)	4 (2)	
>1 cancellation **[n (%)]**	78 (49)	33 (21)	17(11)	16(10)	13(8)	2(1)	0.73
**How angry were you? [n (%)]**	107 (32)	41 (12)	45 (13)	47 (14)	88 (26)	10 (3)	
1 cancellation **[n (%)]**	51 (29)	24 (13)	25 (14)	24 (13)	50 (28)	4 (2)	
>1 cancellation **[n (%)]**	56 (35)	17 (11)	20 (13)	23 (14)	38 (24)	6 (4)	0.69
**How frustrated were you? [n (%)]**	139 (41)	58 (17)	42 (12)	52 (15)	36 (11)	12 (4)	
1 cancellation **[n (%)]**	70 (39)	36 (20)	20 (11)	30 (17)	19 (11)	5 (3)	
>1 cancellation **[n (%)]**	69 (43)	22 (14)	22 (14)	22 (14)	17 (11)	7 (4)	0.57
**How stressed were you? [n (%)]**	129 (38)	49 (14)	41 (12)	42 (12)	71 (21)	7 (2)	
1 cancellation **[n (%)]**	67 (36)	28 (15)	22 (12)	26 (14)	41 (22)	2 (1)	
>1 cancellation **[n (%)]**	62 (41)	21 (14)	19 (12)	16 (10)	30 (20)	5 (3)	0.61

For peer review only

Table 3: Sample of free text comments from responders

"I was aware of winter pressures and was expecting op to be cancelled. Felt it was right someone with greater need took priority."
"As I am on my own, with no family nearby, it had no real impact. Just a few phone calls to cancel lifts in and back. New appointment for 4th December, so not long to wait. Quite understood situation with the start of the "winter strain", so no point in getting stressed. No complaints at all."
"Struggling to swallow, risk of choking when eating."
"The surgeon was ready to do my operation on the correct day but was told at the last minute there was no bed for me in the ICU/high dependency ward so he couldn't do it. I was very disappointed, but it was not the surgeon's fault."
"I totally understood why my operation was cancelled. My operation needed to be done but my life was not in danger. It was just keeping my work informed so cover could be found whilst I was off work"
"It was cancelled the day before. This meant I had to re-arrange work, so did my partner and the childcare for 2 days. All at late notice. We all work full time. I was also due to fly to America for work and this now became an issue."
"I was disappointed on the original date being cancelled and postponed by 9/10 weeks. It was not a very serious lift threatening situation for me and there were other serious cancer operations that required operations so I fully understood the reasons etc."

For peer review only

"Obviously at the time I felt sad because you not only gear yourself up to have the operation, but I could not justify taking up a hospital bed when there were more needy cases than mine requiring treatment. I do feel more money and less stress for NHS workers is required and the government needs to take heed!"

"I was waiting 6 months for this operation. This meant our lives were "put on hold" for this time. We could not book holidays, arrange anything that involved being away from home as we were expecting op dates; no information on delay times was given. This is very stressful"

"I was surprised to be given pre-med drugs before being told the operation was cancelled. I left the hospital feeling "spaced out"!"

"Each time I came for pre op washout the day before to be told when called in that there is no operation tomorrow. I have a young family so I have to prepare them emotionally."

"I am usually active and my hobbies and enjoyment usually is by way of playing sport. Not being able to undertake these activities had the obvious physical affects and resulted in me being in a low mood, even feeling depressed at times. In turn, I found myself comfort eating and drinking more alcohol. In conclusion, the physical and mental effects were profound."

"Operation performed 5 weeks later, did not affect outcome."

"Cancelled holiday. Developed sepsis after being discharged."

"Very disappointed."

"Such late notice when each operation was cancelled (day before). Very mentally draining preparing for our op then it getting cancelled."

For peer review only

"I was given a date. When I reached hospital there was no bed. Cancellation happened three times"

"My operation was cancelled due to a trauma case on that day. Everyone at the hospital was so kind and op was arranged for the next week. Thank you."

"My biggest problem has been the effect the original cancellation had on my elderly mother (aged 97) who was booked into respite care for six weeks @ £500 per week. She is still there some six months later. Her chances of coming home have diminished to almost none."

"It was rubbish at the time but completely understandable. Government should take the hit not the NHS. You guys are great and do all you can!"

"I was very distressed, I cried frequently and was very anxious. I felt let down and became depressed. I have not felt like doing anything and have rarely left the house. My irritable bowel syndrome got worse and my mental health has not been good and I have to take medication. My relationships have become strained and I feel I can't trust people."

"It made me feel that I didn't really matter! Although my condition is not life threatening, it is life debilitating."

For peer review only

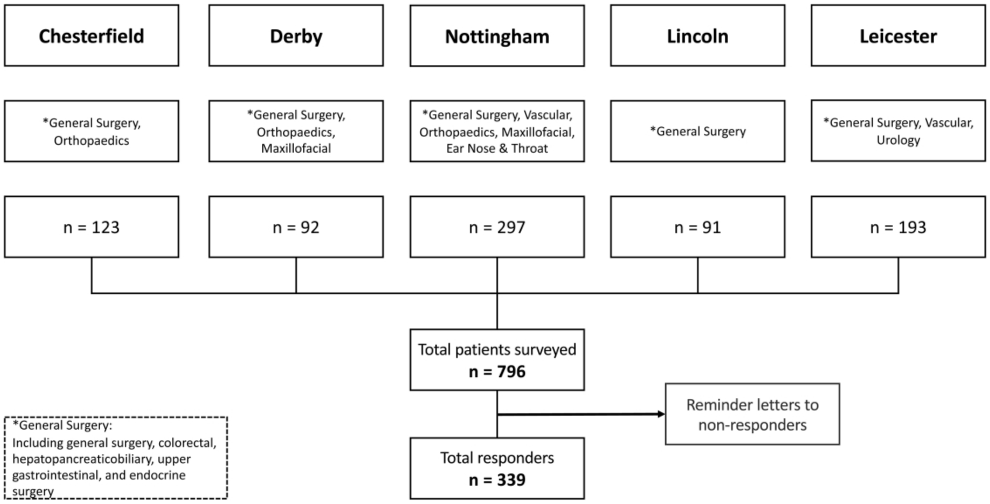

Figure 1: Study flow diagram.

282x144mm (300 x 300 DPI)

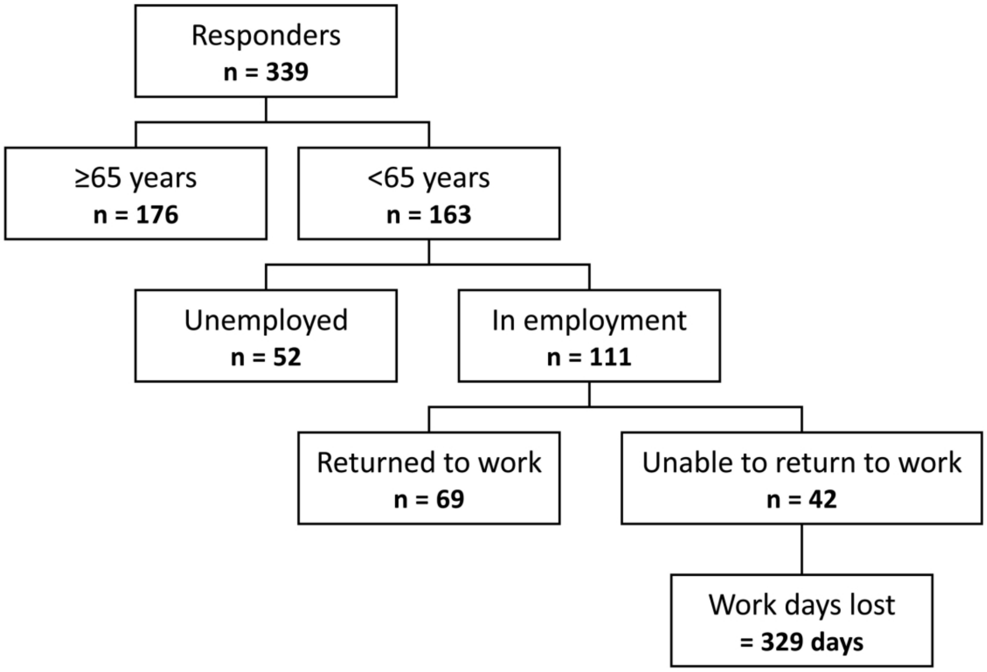

Figure 2: Number of work days lost.

188x129mm (300 x 300 DPI)

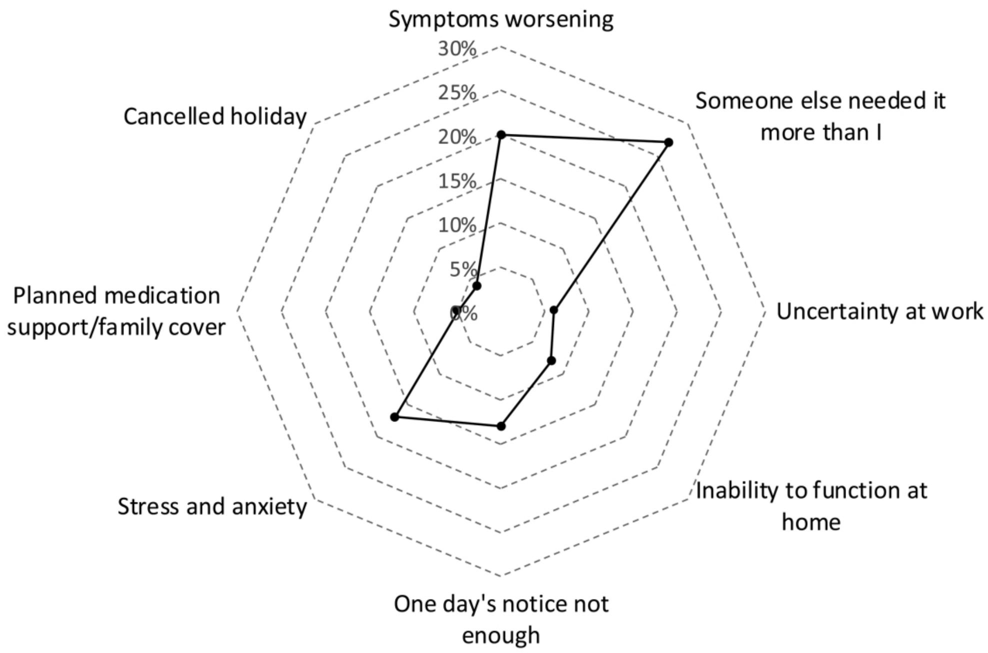

Figure 3: Radar diagram of thematic analysis (figures represent percentage of responders).

231x150mm (300 x 300 DPI)

Winter Elective Surgery Cancellation and Psychological Impact survey

WES-Pi

Please tell us about you:

Q1) What is your employment status?
*Please select **one** option only.*

Employed	- full time	☐	In education	- full time	☐
	- part time	☐		- part time	☐
Self-employed	- full time	☐	Unemployed		☐
	- part time	☐	Retired		☐

Q2) What is your age group?
*Please select **one** option only.*

16-24 years	☐	65-74 years	☐
25-34 years	☐	75-84 years	☐
35-44 years	☐	Over 85 years	☐
45-54 years	☐		
55-64 years	☐	Prefer not to say	☐

Q3) On what date was your operation meant to be?
Please provide an estimate if you are unsure of the exact date.

Date: d d - m m - y y y y

Q4) On what date were you told your operation was cancelled or postponed?
Please provide an estimate if you are unsure of the exact date.

Date: d d - m m - y y y y

Q5) If your operation was cancelled or postponed previously, how many times did this occur in total?
Please provide an estimate if you are unsure of the exact number of times.

[ ] times

For peer review only - http://bmjopen.bmj.com/site/about/guidelines.xhtml

Q6) What operation were you meant to be having?

Please describe in your own words, e.g. hernia operation, hip replacement, etc.

Please tell us about the impact of your operation being cancelled or postponed on your work and finances:

Q7) Due to your operation being cancelled or postponed, were you able to **go to work** instead?

Please provide an estimate of the days you think you needed to take off work.

Yes ☐
No* ☐
Not applicable ☐

*If not, how many **days off** did you take?

Q8) Do you think you will need **extra days off work** because your operation was cancelled or postponed?

Please provide an estimate of the day/s you think you will need off work, due to your untreated condition preventing you from working, or the additional time off you will have to take when you receive another date for your operation.

Yes* ☐
No ☐
Not applicable ☐

*If yes, how many **day/s off**?

Q9) Did your friends or family members have to take **extra days off work** because your operation was cancelled or postponed?

Please provide an estimate of the day/s your friends or family members have taken so far, to either look after you when unwell (because you did not have your operation), or planned to take off to look after you following your operation

Yes* ☐
No ☐
Not applicable ☐

*If yes, how many **day/s off**?

For peer review only - http://bmjopen.bmj.com/site/about/guidelines.xhtml

For peer review only

Q10) If you are a parent or carer, have you needed **extra childcare** or **carer support**, because your operation was cancelled or postponed?

Please provide an estimate of the total time you have needed so far. e.g. having to book extra support when you are unwell (because you did not have your operation), or needing to rebook extra support for when you receive another operation date.

Yes* ☐
No ☐
Not applicable ☐

*If yes, how many **hours*** or **days***?
**please circle as appropriate*

Q11) Have you had **extra costs in travel and parking** because your operation was cancelled or postponed?

Please provide an estimate if you are unsure of the exact costs so far. e.g. additional journeys to the hospital/doctors because you have been unwell (because you did not have your operation), or because you have needed extra tests before receiving another operation date.

Yes* ☐
No ☐
Not applicable ☐

If yes, how much **extra cost**?

Please tell us about the impact of your operation being cancelled or postponed on your mental health and well-being:

Q12) Are there any key **social activities/hobbies** you have had to miss because your operation was cancelled or postponed?

These may include family days out, attendance at weddings, visiting friends, or your ability to physically participate in your hobbies or interests outside of work.

Yes* ☐
No ☐
Not applicable ☐

*If yes, is there anything specific you would like to mention?

For peer review only - http://bmjopen.bmj.com/site/about/guidelines.xhtml

For each question please select **one** option that best describes **your feelings, <u>at the time</u>** your operation was cancelled or postponed:

	Not at all	Slightly	Moderately	Very	Extremely	Not applicable
Q13) How **sad** were you?	☐	☐	☐	☐	☐	☐
Q14) How **disappointed** were you?	☐	☐	☐	☐	☐	☐
Q15) How **angry or annoyed** were you?	☐	☐	☐	☐	☐	☐
Q16) How **frustrated** were you?	☐	☐	☐	☐	☐	☐
Q17) How **stressed** were you?	☐	☐	☐	☐	☐	☐

For each question please select **one** option that best describes **your concerns, <u>at the time</u>** your operation was cancelled or postponed:

	Not at all	Slightly	Moderately	Very	Extremely	Not applicable
Q18) How concerned were you about your **symptoms continuing**?	☐	☐	☐	☐	☐	☐
Q19) How concerned were you about your **health deteriorating**?	☐	☐	☐	☐	☐	☐

Q20) Is there anything else you would like to mention about how your operation being cancelled or postponed has affected you?
e.g. how it made you feel, how it impacted on your life, how it affected you physically, whether you were given enough information, etc.

Thank you for taking the time to complete this survey

Please return it in the pre-paid envelope provided within **<u>two weeks</u>** of receipt.

CODE: [ ]

For peer review only - http://bmjopen.bmj.com/site/about/guidelines.xhtml

National Institute for Health Research

WES-Pi

1st March 2018

Dear Patient,

As you may be aware, due to the increased pressures faced by the NHS during the winter period, some patients like yourself have unfortunately had their operations either cancelled or delayed.

To understand the true impact this can have on our patients' lives, we are asking all patients who have experienced a cancellation or delay to complete the attached short survey (Winter Elective Surgery Cancellation and Psychological Impact survey).

You do not have to participate in this survey, but your experiences may help us to improve our services for future patients. There are no right or wrong answers to any of the questions asked, it is your opinions that we are really interested in. In particular, we would like to hear how your experience of a cancellation has affected you emotionally, physically, and financially.

You do not have to give any personal details, and the results of the survey will be completely anonymous when published. To participate, please fill in the attached survey and return it to us in the pre-paid envelope provided. If you have any further questions about this survey you can contact us at: wespistudy@gmail.com

Thank you for your time.

Yours sincerely,

The East Midlands Surgical Academic Network (EMSAN)

EMSAN is a group of trainee doctors who work for the NHS. We believe in making research opportunities available to everyone, to aid learning and improve medical services.

Visit us at: emsan.co.uk/wespi
Contact us at: wespistudy@gmail.com

For peer review only

Patient Public Involvement in study survey design

Question No.	Question	PPI Lay Assessors – random comments	Study lead response
Q5	In your own words, what operation were you meant to be having?	Wording change: "Had your operation been previously cancelled or postponed? If so, how many times?"	Amended
Q8	How many **extra** days off work do you now think **you** will miss because of the health condition that you were due to have the operation for?	This is a rhetorical question I doubt even Mr Adiamah could answer correctly.	This has now been amended to reflect the time taken for the surgery and immediately afterwards, and potential time taken off due to ongoing illness related to the cancellation
Q10	How much extra cost has **childcare** cost you because of your operation being cancelled or postponed?	Question on childcare costs. May not be relevant to all participants, and potentially quite intrusive. Also, costs can be very variable.<br><br>Suggestion of using 'hours of childcare' instead.	Question amended to ask about 'hours of childcare<br><br>AND carer support' if relevant.<br><br>Descriptor added.
Q13-Q18	Please answer the following questions in relation to your operation being cancelled or postponed.<br><br>*For each question please select ONE option that*	Surely 'today' might not be a good measure of feelings about the change in plan	Amended to ask about feelings at the time of the postponement instead of 'today'.

For peer review only

	best describes your feelings ***today.***<br><br>How **disappointed (Q13); angry (Q14); frustrated (Q15); stressed (Q16)** are you about your operation?<br><br>How concerned are you about<br><br>your **symptoms continuing**? (Q17); **health deteriorating**? (Q18)		
Overall	Additional box for other comments (this was suggested by two of the PPI Lay Assessors)		Great suggestion, thanks – we have added this.

For peer review only

STROBE Statement—checklist of items that should be included in reports of observational studies

	Item No	Recommendation	Page number
**Title and abstract**	1	(*a*) Indicate the study's design with a commonly used term in the title or the abstract	1
		(*b*) Provide in the abstract an informative and balanced summary of what was done and what was found	3
**Introduction**			
Background/rationale	2	Explain the scientific background and rationale for the investigation being reported	6-7
Objectives	3	State specific objectives, including any prespecified hypotheses	7
**Methods**			
Study design	4	Present key elements of study design early in the paper	8-12
Setting	5	Describe the setting, locations, and relevant dates, including periods of recruitment, exposure, follow-up, and data collection	8
Participants	6	(*a*) *Cohort study*—Give the eligibility criteria, and the sources and methods of selection of participants. Describe methods of follow-up<br>*Case-control study*—Give the eligibility criteria, and the sources and methods of case ascertainment and control selection. Give the rationale for the choice of cases and controls<br>*Cross-sectional study*—Give the eligibility criteria, and the sources and methods of selection of participants	8
		(*b*) *Cohort study*—For matched studies, give matching criteria and number of exposed and unexposed<br>*Case-control study*—For matched studies, give matching criteria and the number of controls per case	
Variables	7	Clearly define all outcomes, exposures, predictors, potential confounders, and effect modifiers. Give diagnostic criteria, if applicable	8-12
Data sources/ measurement	8*	For each variable of interest, give sources of data and details of methods of assessment (measurement). Describe comparability of assessment methods if there is more than one group	*8-12*
Bias	9	Describe any efforts to address potential sources of bias	9-12
Study size	10	Explain how the study size was arrived at	8
Quantitative variables	11	Explain how quantitative variables were handled in the analyses. If applicable, describe which groupings were chosen and why	12
Statistical methods	12	(*a*) Describe all statistical methods, including those used to control for confounding	12
		(*b*) Describe any methods used to examine subgroups and interactions	12
		(*c*) Explain how missing data were addressed	12
		(*d*) *Cohort study*—If applicable, explain how loss to follow-up was addressed<br>*Case-control study*—If applicable, explain how matching of cases and controls was addressed<br>*Cross-sectional study*—If applicable, describe analytical methods taking account of sampling strategy	8-12
		(*e*) Describe any sensitivity analyses	NA

For peer review only - http://bmjopen.bmj.com/site/about/guidelines.xhtml

For peer review only

Continued on next page

**Results**			**Page number**
Participants	13*	(a) Report numbers of individuals at each stage of study—eg numbers potentially eligible, examined for eligibility, confirmed eligible, included in the study, completing follow-up, and analysed	13
		(b) Give reasons for non-participation at each stage	13
		(c) Consider use of a flow diagram	Fig1
Descriptive data	14*	(a) Give characteristics of study participants (eg demographic, clinical, social) and information on exposures and potential confounders	13
		(b) Indicate number of participants with missing data for each variable of interest	13
		(c) *Cohort study*—Summarise follow-up time (eg, average and total amount)	
Outcome data	15*	*Cohort study*—Report numbers of outcome events or summary measures over time	
		Case-control study—Report numbers in each exposure category, or summary measures of exposure	*13*
		Cross-sectional study—Report numbers of outcome events or summary measures	
Main results	16	(*a*) Give unadjusted estimates and, if applicable, confounder-adjusted estimates and their precision (eg, 95% confidence interval). Make clear which confounders were adjusted for and why they were included	13-15
		(*b*) Report category boundaries when continuous variables were categorized	
		(*c*) If relevant, consider translating estimates of relative risk into absolute risk for a meaningful time period	
Other analyses	17	Report other analyses done—eg analyses of subgroups and interactions, and sensitivity analyses	13-15
**Discussion**			
Key results	18	Summarise key results with reference to study objectives	16-19
Limitations	19	Discuss limitations of the study, taking into account sources of potential bias or imprecision. Discuss both direction and magnitude of any potential bias	18
Interpretation	20	Give a cautious overall interpretation of results considering objectives, limitations, multiplicity of analyses, results from similar studies, and other relevant evidence	16-19
Generalisability	21	Discuss the generalisability (external validity) of the study results	16-19
**Other information**			
Funding	22	Give the source of funding and the role of the funders for the present study and, if applicable, for the original study on which the present article is based	N/A

*Give information separately for cases and controls in case-control studies and, if applicable, for exposed and unexposed groups in cohort and cross-sectional studies.

Note: An Explanation and Elaboration article discusses each checklist item and gives methodological background and published examples of transparent reporting. The STROBE checklist is best used in conjunction with this article (freely available on the Web sites of PLoS Medicine at http://www.plosmedicine.org/, Annals of Internal Medicine at http://www.annals.org/, and Epidemiology at http://www.epidem.com/). Information on the STROBE Initiative is available at www.strobe-statement.org.

For peer review only - http://bmjopen.bmj.com/site/about/guidelines.xhtml

For peer review only
